# D^2^ Plot, a Matrix of DNA Density and Distance to Periphery, Reveals Functional Genome Regions

**DOI:** 10.1002/advs.202202149

**Published:** 2022-08-30

**Authors:** Yizhuo Che, Xiaofei Yang, Peng Jia, Tingjie Wang, Dan Xu, Tianxue Guo, Kai Ye

**Affiliations:** ^1^ School of Automation Science and Engineering Faculty of Electronic and Information Engineering Xi'an Jiaotong University Xi'an Shaanxi 710049 China; ^2^ MOE Key Lab for Intelligent Networks and Networks Security Faculty of Electronic and Information Engineering Xi'an Jiaotong University Xi'an Shaanxi 710049 China; ^3^ School of Computer Science and Technology Faculty of Electronic and Information Engineering Xi'an Jiaotong University Xi'an Shaanxi 710049 China; ^4^ Key Laboratory of Biomedical Information Engineering of the Ministry of Education School of Life Sciences and Technology Xi'an Jiaotong University Xi'an Shaanxi 710049 China; ^5^ School of Life Science and Technology Xi'an Jiaotong University Xi'an Shaanxi 710049 China; ^6^ Faculty of Science Leiden University Leiden 2300 The Netherlands; ^7^ Genome Institute The First Affiliated Hospital of Xi'an Jiaotong University Xi'an Shaanxi 710049 China

**Keywords:** 3D genome, DNA density, nuclear lamina, single‐cell Hi‐C

## Abstract

The execution of biological activities inside space‐limited cell nuclei requires sophisticated organization. Current studies on the 3D genome focus on chromatin interactions and local structures, e.g., topologically associating domains (TADs). In this study, two global physical properties: DNA density and distance to nuclear periphery (DisTP), are introduced and a 2D matrix, D^2^ plot, is constructed for mapping genetic and epigenetic markers. Distinct patterns of functional markers on the D^2^ plot, indicating its ability to compartmentalize functional genome regions, are observed. Furthermore, enrichments of transcription‐related markers are concatenated into a cross‐species transcriptional activation model, where the nucleus is divided into four areas: active, intermediate, repress and histone, and repress and repeat. Based on the trajectories of the genomic regions on D^2^ plot, the constantly active and newly activated genes are successfully identified during olfactory sensory neuron maturation. The analysis reveals that the D^2^ plot effectively categorizes functional regions and provides a universal and transcription‐related measurement for the 3D genome.

## Graphical abstract

The D^2^ algorithm is developed to detect two physical properties, DNA density and distance to nuclear periphery, from the reconstructed genome structures. Based on them, D^2^ plot is constructed to reveal the spatial positions of genetic and epigenetic markers. Furthermore, a cross‐species transcriptional activation model on D^2^ plot is summarized, which is able to reflect expression levels.

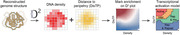

## 1 Introduction

DNA molecules are highly condensed inside cell nuclei to compact into limited space, with the necessary loci exposed for active transcription.^[^ ^1^ ^]^ The rapid development of 3C‐based technologies offers insight into the sophisticated organization of the genome, allowing for the discovery of functional nuclear structures, for example, TADs, loops, and compartments.^[^ ^2^ ^]^ Nevertheless, most of these interaction‐derived structures focus on local spatial organizations, lacking a universal ability to compare distal regions. Although compartments are genome‐wise structures, they fail to correlate a given region to a specific biological function without additional functional data.^[^ ^2^ ^]^ While some researchers use interdisciplinary approaches to delineate the chromatin structure,^[^ ^3^, ^4^, ^5^ ^]^ their methods continue to focus on interactions. Therefore, universal measurement for a 3D genome with functional correlation is required to elucidate the structural basis of genome function.

DNA density (hereafter referred to as density) and distance to nuclear periphery (DisTP) are two primary physical properties that are related to transcriptional activities in genome spatial organization. A negative correlation between density and transcriptional activity has been implied by several nuclear structures in previous studies.^[^ ^6^, ^7^, ^8^ ^]^ Specifically, the densely packed heterochromatin is less accessible and transcriptionally repressed,^[^ ^6^, ^9^ ^]^ whereas euchromatin, which is less condensed, exhibits more active transcription.^[^ ^6^ ^]^ Several studies have shown that DisTP is positively correlated with transcriptional activity.^[^ ^9^, ^10^, ^11^, ^12^ ^]^ The lamina‐associated domains (LADs), located at the nuclear periphery, are believed to repress gene expression.^[^ ^9^ ^]^ Experimental evidence has reinforced the idea that reduced proximity to the nuclear lamina is usually accompanied with higher transcriptional activity, while increased proximity to the nuclear lamina results in more repressed transcription.^[^ ^13^, ^14^ ^]^ Previous works have clustered the genomic segments into groups with different DisTP (1 Mb resolution), either according to the results of experimentation^[^ ^11^ ^]^ or those of simulation.^[^ ^12^ ^]^ By computing the correlation with genetic markers, such as histone modifications, they found that the inner groups were more likely to enrich for active markers compared with the peripheral groups.

Owing to the lack of appropriate techniques for detecting the physical properties in genome‐wide scale with high resolution, the extent to which density or DisTP are connected to transcriptional activities, and the analysis comprising the properties for the comprehensive description of genome structure and function are unclear. We developed an algorithm, D^2^, to compute the two properties: density and DisTP, and integrated them as a 2D matrix, D^2^ plot, for mapping functional markers at the genome‐wide scale. We identified distinct enrichment patterns of functional markers on the D^2^ plot and proposed a cross‐species transcriptional activation model on D^2^ plot. Our study revealed a novel analysis procedure for correlating nuclear organization with gene transcriptional activities and provided a new perspective for the study of the 3D genome structure.

## 2 Results

### 2.1 Detection of DNA Density and DisTP

**Figure 1:**
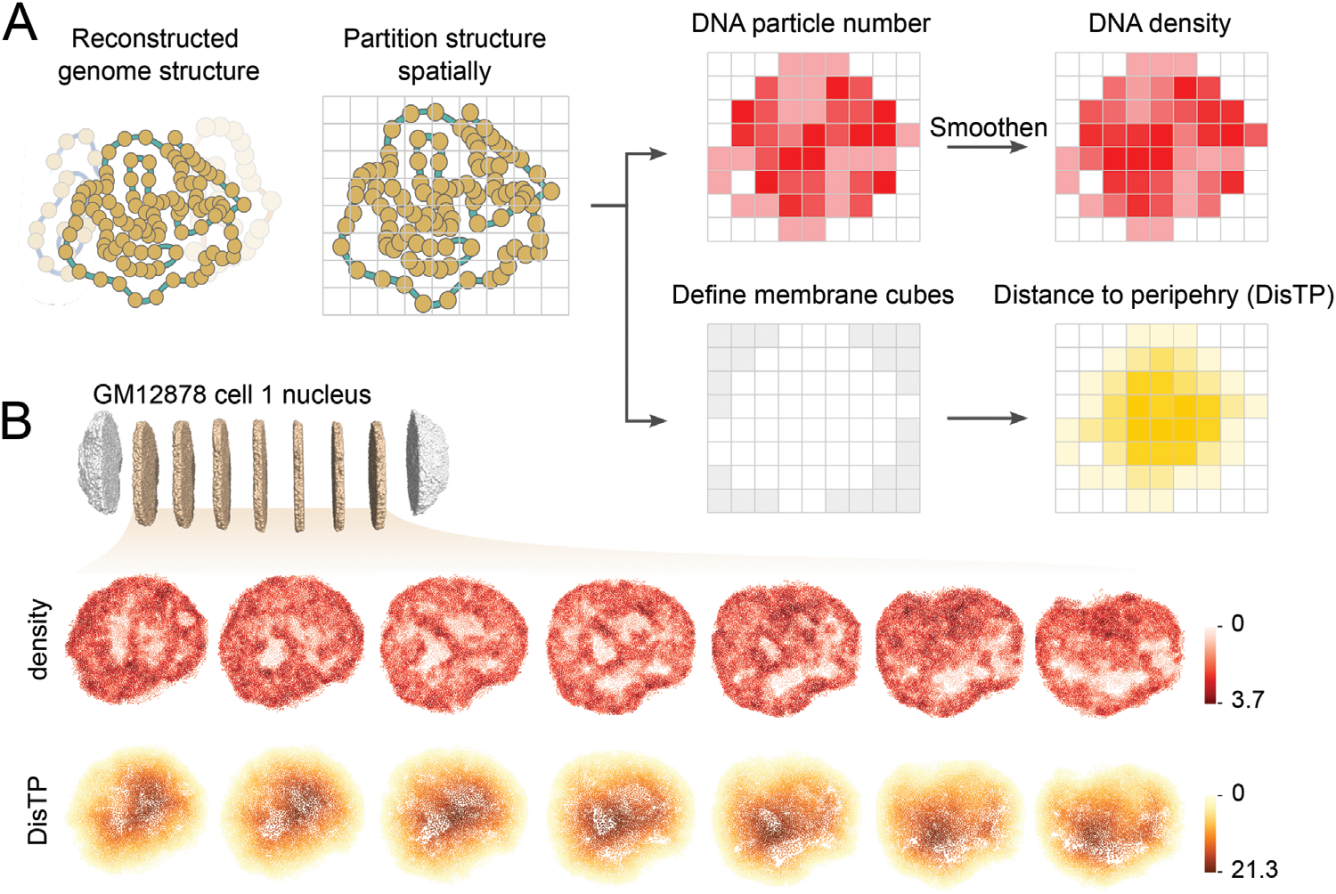
D^2^ detects DNA density and DisTP at a genome‐wide scale. A) Illustration of the D^2^ algorithm. Each yellow dot from the reconstructed structure represents a 20 kb genomic segment. The line threading yellow dots indicates they are part of a given chromosome. The light gray mesh is the 3D segmentation, which is shown in 2D for clear visualization. In the “define membrane cubes” panel, the gray cubes are regarded as the membrane cubes as they are not buried. The intensity of red for each cube in the right top subpanels indicates its density value, while the intensity of yellow in the right bottom subpanel indicates its DisTP value. B) Serial sections of a sample nucleus (GM12878 cell 1) showing density (red) and DisTP (yellow). Only seven sections in the middle are shown. Each section is 8% of the entire width. The white patches on the sections denote the empty or almost empty regions inside the nuclei.

D^2^ algorithm computed **D**ensity and **D**isTP at the genome‐wide scale in high resolution (**Figure** **1**A; the Experimental Section). The input of the D^2^ algorithm was a set of DNA particles, which were reconstructed from the single‐cell Hi‐C contact map by Dip‐C^[^ ^15^ ^]^ or NucDynamics^[^ ^16^ ^]^ (Table S1, Supporting Information; the Experimental Section). Each particle had its own spatial coordinate and genomic position, thus linking spatial structure to genomic sequence. First, we introduced a 3D mesh segmentation to assign the input DNA particles into different cubes. The cube length was adjusted so that the average number of particles for each cube was approximately equal among cells, thereby allowing for intercell comparison. Density was calculated as the number of particles in one cube and then smoothed to reduce noise. To obtain DisTP, we first identified the membrane cubes (cubes located at the nuclear periphery), and then calculated DisTP for each cube as its distance to the nearest membrane cubes (Figure S1, Supporting Information). Both properties were consistent across different reconstructed replicates of a given individual cell (Figure S2, Supporting Information), indicating that D^2^ was robust against the randomness introduced by the structure reconstruction algorithm. Therefore, we selected the first reconstruction of each cell for the following analysis. Serial sections of density and DisTP of a sample cell were illustrated in Figure 1B.

**Figure 2:**
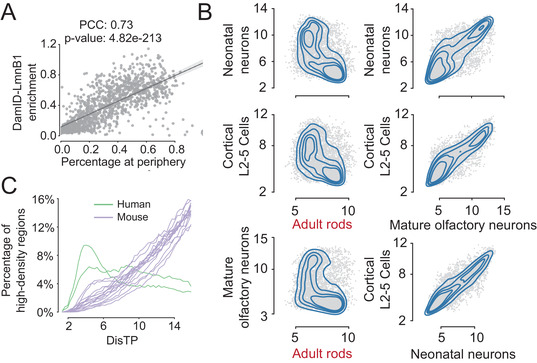
Validation of D^2^ algorithm. A) Scatter plot of the experimental LAD data versus the probability of periphery regions from the D^2^ algorithm. Each point denotes a 2 Mb DNA segment of the mESC cell. The correlation and significance are computed by Pearson correlation (*n* = 1103). The black line shows the fitting line of linear regression. B) Correlations of computed DisTP among different cell types. Each scatter plot includes 50 000 randomly sampled 20 kb genomic segments. Detailed analysis of adult rods is shown in the Supporting Information. C) Percentage of high‐density regions for a set of given DisTP. Each line denotes a cell type. The top 5% of dense regions are regarded as the high‐density regions.

We then validated the results of D^2^ with experimental evidence. LADs were the conspicuous nucleic structures that were mainly located at the nuclear periphery. We found a significantly high correlation between the probability of appearing at the nuclear periphery (low DisTP) and the experimental LAD data, thereby verifying the fitness of DisTP (**Figure** **2**A; Pearson coefficient correlation (PCC): 72.87%, *p*‐value: 4.8 × 10^−213^). Rods of nocturnal animals adopted an inverted radial organization, in which euchromatin resided at periphery and heterochromatin preferred the interior.^[^ ^17^ ^]^ Inverted distributions between the computed DisTP of rods and that of other cell types were observed (Figure 2B), indicating that our algorithm is able to capture diverse configurations of DisTP from dramatically different genome structures. Owing to the lack of experimental measurement for density, we attempted to validate this through highly compacted chromocenters located at the inner nuclei of mouse cells but not in human cells (Figure S3, Supporting Information).^[^ ^18^, ^19^ ^]^ We found that high‐density regions preferentially located at the inner nuclei of mouse cells but this was not the case in human cells (Figure 2C), indicating the feasibility of density detection.

**Figure 3:**
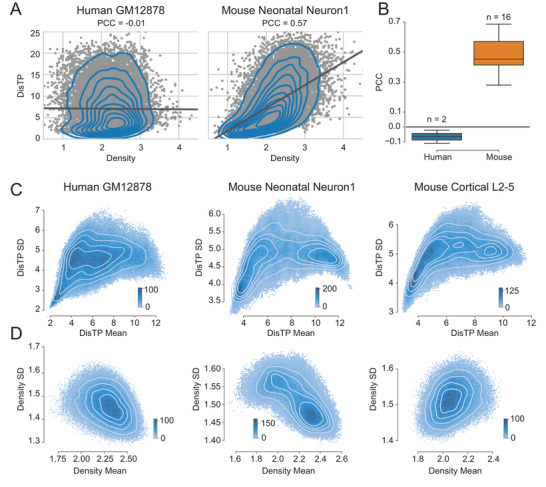
Strong intrinsic stochasticity of density and DisTP. A) Scatter and kernel density estimate (KDE) plots of density and DisTP. The gray line delineates the fitting line of linear regression. The correlations are computed by Pearson correlation. Detailed information for the additional 15 cell types is shown in Table S2 of the Supporting Information. B) Boxplot of PCC between density and DisTP for human and mouse cells. *n* indicates the number of cell types. C) 2D histogram and KDE plots of mean and SD for DisTP. One human and two mouse cell lines are shown here, and additional 14 cell types are shown in Figure S5 of the Supporting Information. D) Similar to (C) but for density.

We then investigated the correlation between density and DisTP. In human cells, density and DisTP were largely independent of each other (PCC: −0.019 and −0.103; **Figure** **3**A,B; Table S2, Supporting Information). By contrast, they were positively correlated in mouse cells (PCC: 0.278 to 0.673, Figure 3A,B; Table S2, Supporting Information). This interspecies difference indicated that human and mouse cells adapted different strategies for nuclear spatial organization.

Next, we explored the stochasticity of density and DisTP by computing their standard deviations (SDs; Figure S4, Supporting Information; the Experimental Section). SDs of density (≈1.5) and DisTP (≈4.7) were comparable to their means (≈2.2 and ≈6.8, Table S3, Supporting Information), indicating a strong intrinsic stochasticity that was consistent with previous observations.^[^ ^20^ ^]^ In addition, certain regions with specific density or DisTP had lower standard deviation (SD). Specifically, for DisTP, the periphery regions showed lower SD for both human and mouse cells (Figure 3C; Figure S5A, Supporting Information), thereby coinciding with their roles as anchors for attachment to the nucleus membrane. For density, the lower SD regions exhibited noticeable cell‐type specificity. For most cell types (11 out of 17), high‐density regions were lower in SD, while for the other six mouse neuron cell types, low‐density regions had lower SD (Figure 3; Figures S5B and S6, Supporting Information), suggesting a cell‐type‐specific anchoring mechanism for density.

### 2.2 Distinct Patterns of Functional Markers on D^2^ Plot Revealed Functional Genomic Regions

The spatial organization of eukaryotic cell nuclei plays a pivotal role in regulating the biological activities of DNA molecules.^[^ ^1^, ^21^, ^22^ ^]^ However, a comprehensive and systematic analysis of the correlation of density and DisTP with chromatin activities was lacking. To achieve that, we collected different types of epigenomic and genomic data from human GM12878 cells and mouse neonatal forebrain neurons, including DNA methylations, histone modifications, transcription factor binding sites, genomic repeats, and chromatin states.

**Figure 4:**
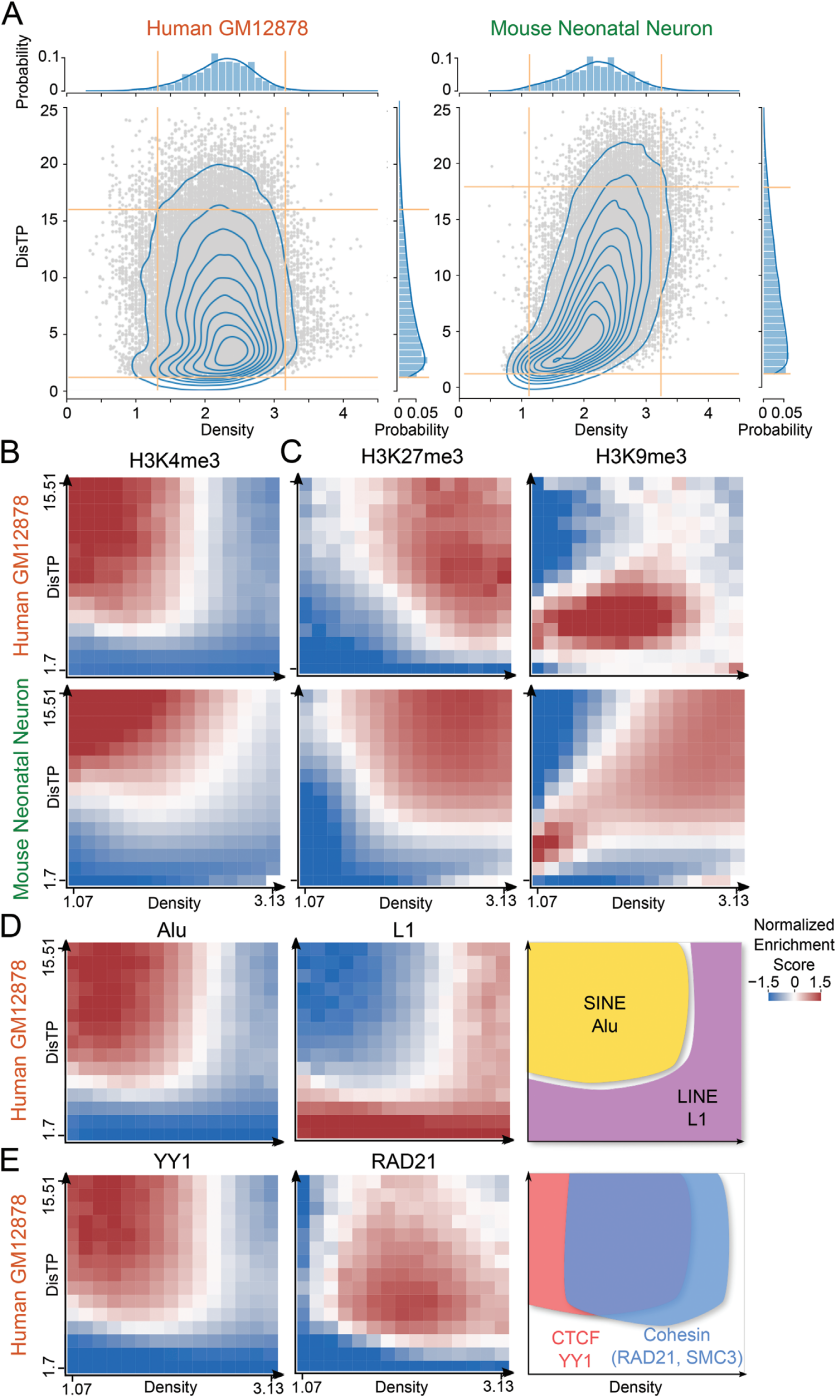
Enrichment scores of genetic and epigenetic markers on D^2^ plot. A) 2D histogram and KDE plots of density and DisTP in human GM12878 cells and mouse neonatal forebrain neurons. The top and right subpanels show the 1D histograms of density (top) and DisTP (right). Yellow lines mark the boundaries to exclude outliers from downstream analysis. The boundaries of 16 cell types are shown in Table S4 of the Supporting Information. B) Enrichment scores of H3K4me3 marker of human and mouse cells on D^2^ plot. The enrichment score for the bottom right physical state in mouse cells is not shown owing to its small number (*n* < 200) of genomic segments (Methods section, Supporting Information). C) Similar to (B) but with markers H3K27me3 and H3K9me3. D) Enrichment scores of Alu and L1 for human GM12878 cell line. The results for mouse cell lines are shown in Figure S11 of the Supporting Information. The right panel illustrates the competitive relationship between Alu and L1 in both human and mouse cells. E) Enrichment scores of YY1 and RAD21 for human GM12878 cell line. The enrichment scores of CTCF and SMC3 are shown in Figure S13 of the Supporting Information. The right panel illustrates the mostly cooperative but occasionally independent relationships among CTCF, YY1, and cohesin. Details on the process of computing enrichment scores are presented in Methods section of the Supporting Information.

For joint analysis, we constructed a 2D matrix of density (*x*‐axis) and DisTP (*y*‐axis), named D^2^ plot (the Experimental Section). D^2^ plot was composed of physical states (the Experimental Section), the rectangular bins with specific values of density and DisTP (**Figure** **4**A; Table S4, Supporting Information). We only kept the states with enough genomic segments for statistical significance. Genetic markers were then mapped to the D^2^ plot according to their enrichments on genomic segments. Enrichment scores of each marker at each physical state were calculated, compared to the whole genome as the background (the Experimental Section). The enrichment scores of all markers are shown in Figure 4 and the figures in the Supporting Information.

We first examined the distribution of active markers on D^2^ plot. We found that H3K4me3, a well‐established marker for transcriptional activation,^[^ ^23^ ^]^ enriched at the center rather than at the periphery of the nuclei, which was consistent with previous microscopic observations (Figure 4B; Figure S7, Supporting Information).^[^ ^7^ ^]^ Regions with enriched H3K4me3 distribution showed lower density (Figure 4), which confirmed that transcriptional regulators around active genes lowered the local DNA density.^[^ ^6^ ^]^ Other active histone markers and chromatin states, and even the poised or weakly transcribed chromatin states exhibited similar patterns (Figure S8, Supporting Information). The coexistence of active and weak transcriptional markers implied that the inner low‐density areas were not a determining factor for transcriptional activity, but rather provided a hospitable environment, allowing for the recruitment of regulating proteins for transcriptional activation, and thereby reinforcing the previously proposed “permissive role” of spatial organization.^[^ ^24^ ^]^

Next, we focused on the facultative and constitutive heterochromatins with repressed transcription, as marked by the trimethylation of lysine 27 on histone H3 (H3K27me3) and trimethylation of lysine 9 on histone H3 (H3K9me3), respectively.^[^ ^25^ ^]^ On the D^2^ plot, H3K27me3 was more likely to occupy the condensed regions inside the nuclei, while H3K9me3 showed an obvious preference for nuclear periphery (Figure 4C), as confirmed by the immunofluorescence experiments (Figures S9 and S10, Supporting Information).^[^ ^26^, ^27^ ^]^ We also found that H3K9me3 enriched at inner high‐density regions in mouse cells but not in human cells, suggesting the species‐specific features of heterochromatin arrangements.^[^ ^18^, ^19^ ^]^

We then investigated the repetitive elements on the D^2^ plot. We found that long repeats, including long terminal repeats and long interspersed nuclear elements (for example, L1), preferred the inner high‐density regions or periphery regions (Figure 4D; Figure S11, Supporting Information). Contrarily, the short interspersed nuclear elements (including Alu, B2, and ID) preferably enriched at inner low‐density regions (Figure 4; Figure S10, Supporting Information), which was consistent with the FISH analysis performed by Lu et al., who found that Alu clustered at the nuclear interior, while L1 clustered at the nuclear periphery.^[^ ^28^ ^]^ The distributions of Alu and L1 were significantly negatively correlated in both species (Figure S12, Supporting Information), indicating a competitive relationship between the two transposons in spatial organization.

Next, we examined the distribution of the architectural proteins, including RAD21, SMC3 (both subunits of cohesin), CTCF, and YY1. They are believed to cooperate in the construction of genomic structures via the use of a previously proposed loop extrusion model.^[^ ^29^ ^]^ The patterns of these proteins largely overlapped on the D^2^ plot at the inner region of the nuclei (Figure 4E; Figure S13, Supporting Information). Nonetheless, YY1 and CTCF were more likely to enrich at the lower‐density regions than cohesin, indicating that YY1 and CTCF preferred to locate at the active compartment. Consistently, the fold changes of YY1 (1.71) and CTCF (1.23) enrichments in compartment A to B were higher than those of RAD21 (1.13) and SMC3 (1.15) (Figure S14, Supporting Information). The differences in enrichments implied that, in addition to working together, these proteins might function individually, as previously observed by Wei et al.^[^ ^30^ ^]^

### 2.3 Cross‐Species Transcriptional Activation Model on D^2^ Plot

**Figure 5:**
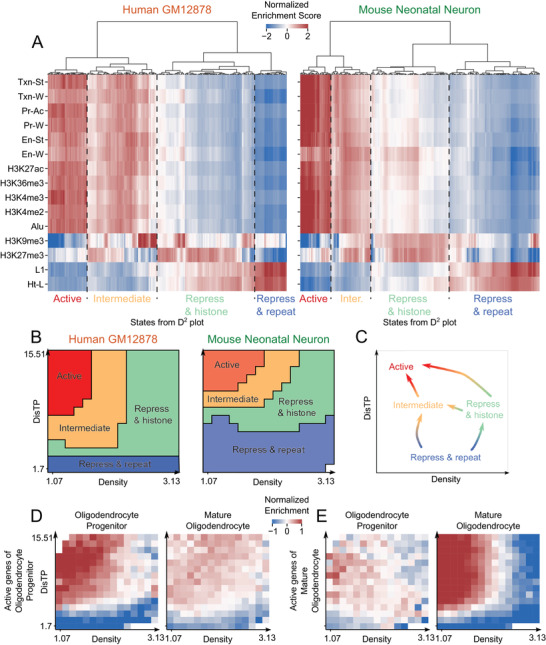
Cross‐species transcriptional activation model on D^2^ plot. A) Hierarchy clustering of D^2^ plot physical states (*n* = 225 for human, *n* = 224 for mouse due to one state with fewer than 200 genomic segments) based on the 16 transcription related markers. Using the optimal ranking hierarchy cluster, the states are ranked automatically according to their activation levels. The number of clusters is set as 4 for both species. The black dashed lines mark the boundaries of the clusters. The abbreviations have been spelled out in the legends of Figures S7 and S10 of the Supporting Information. B) The four areas on each D^2^ plot for either human GM12878 cells or mouse neonatal neurons, corresponding to (A). C) Illustration of the transcriptional activation model on the D^2^ plot. The arrows point to more active regions. D) The enrichment scores of newly activated genes on the D^2^ plots of oligodendrocyte progenitors and mature oligodendrocytes. The enrichment scores of blank physical states are not shown owing to the small number (*n* < 200) of genomic bins. E) Similar to (D) but with newly repressed genes.

To link transcription activity to the D^2^ plot, we selected 16 transcription‐related markers and performed the hierarchy cluster on physical states (the Experimental Section). As shown in **Figure** **5**A, we successfully distinguished the active states from the repressed ones. However, there were still intermediate states marked by both active markers and repressive histone modifications, corresponding to the “bivalent” genomic regions where promoters were prepared for rapid activation during differentiation.^[^ ^31^ ^]^

Based on the hierarchy clusters for both human and mouse cells, we divided the physical states into four groups: active, intermediate, repress and histone (repressed states marked by repressed histone modifications), and repress and repeat (repressed states marked by long repeats) areas. Then we assigned these four areas back to the D^2^ plot to reveal their transcriptional patterns (Figure 5B). The patterns between human GM12878 cells and mouse neonatal forebrain neurons showed slight differences. For example, by computing the property strength (Figure S15, Supporting Information; the Experimental Section), we found that DisTP had less impact on the nucleic interior in human cells compared to mouse cells, probably owing to the different strategies of heterochromatin organization between the two species.^[^ ^18^ ^]^ Other than the marginal difference, the patterns were highly similar, out of which we proposed a cross‐species transcriptional activation model on the D^2^ plot (Figure 5C). The model showed that the most inactive area, repress and repetitive, located at the periphery regardless of density. At the inner nucleus, however, density and DisTP jointly affected gene regulation. From inactive to active areas (repress and histone, intermediate to active area), density decreased while DisTP increased. This model is largely consistent with the current understanding of density and DisTP, but provides additional functional annotation.

To verify the proposed model, we projected the lineage‐specific active genes of mouse brain cells onto the D^2^ plot and found that those active genes located at more interior regions with lower density in the corresponding cell types compared with other cell types (Figure S16, Supporting Information). Next, we focused on the genes that were previously defined as either newly activated or repressed during maturation from oligodendrocyte progenitors to mature oligodendrocytes.^[^ ^32^ ^]^ As expected, the newly activated genes distributed almost randomly on the D^2^ plot of progenitors, but enriched at inner low‐density regions after the maturation (Figure 5D), corresponding to the active and intermediate areas. Conversely, the newly repressed genes located at inner low‐density regions in progenitors but distributed randomly in mature cells (Figure 5E).

Tan et al. found no explanation for the adult‐neuron‐specific genes maintaining the same radial position during differentiation, despite the newly activation of these genes.^[^ ^32^ ^]^ We revealed that DisTP values of these genes remained unchanged (Fisher Test *p*‐value: 0.68), but density values declined significantly (Fisher Test *p*‐value: 3.34 × 10^−148^; Figure S17, Supporting Information), demonstrating that although DisTP alone was not sufficient for predicting gene regulation, its combination with DNA density was instructive.

### 2.4 Trajectories on D^2^ Plots Revealed Transcriptional Activation Modes

After establishing the correlation between the transcriptional level and the D^2^ plot, we aimed to identify the transcriptional patterns of genomic regions merely by their trajectories on the D^2^ plot. To do this, the activation index was introduced as the probability of a given genomic region appearing at the active or intermediate areas, and changes in its value reflected the trajectories on the D^2^ plot during cell maturation (Figure S18, Supporting Information; the Experimental Section). We examined our approach on olfactory sensory neurons (OSNs), which included three cell types at different differentiating stages (newborn, developing, and mature OSNs). By investigating the trajectories of the whole genome on the D^2^ plot, we identified three transcriptional modes: constantly active (CA), repressed to active (RA), and constantly repressed (CR), which revealed the dynamic regulation of gene expression during OSN development.

**Figure 6:**
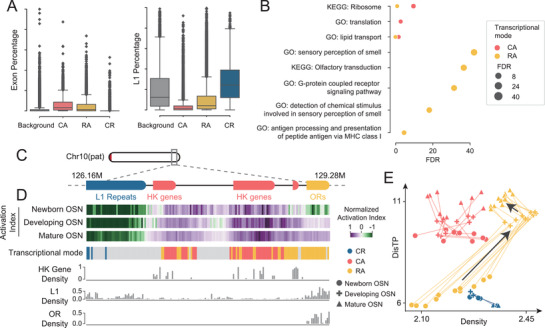
Trajectories on D^2^ plots reveal the transcriptional activation modes. A) Boxplots for percentages of exon (left) and L1 (right) in three identified modes, namely constantly active (CA), repressed to active (RA), and constantly repressed (CR). For background datasets, 20 000 bins from the whole genome are randomly selected. B) Enriched GO terms and KEGG pathways of the three modes. Only the top five significant terms (FDR < 0.05) are plotted. No significant term is found for the CR, therefore it is not shown. C) Schematic of a 3 Mb genomic segment of paternal haplotype containing 19 HK genes, 10 ORs, and 129 L1 repeats. The colored segments mark the locations of these functional elements. D) The top three heatmaps show the activation indexes of three OSN cell types. The three identified modes are shown beneath them. The gray bins are not classified into the three modes. The bottom three bar plots denote the HK gene density, L1 density, and OR density at the 3 Mb genomic region. Each bin at the heatmap or bar plot indicates a 20 kb genomic segment. E) The trajectories of the sample genomic segments on the D^2^ plot. The trajectories for other genomic segments are shown in Figure S20 of the Supporting Information. The black arrows show the directions of trajectories for ORs.

Next, we investigated the enrichments of functional elements in the three modes. Compared to the background, the percentages of exons in CA and RA were remarkably higher, while that of CR was lower (**Figure** **6**A), in accordance with their expression levels. In stark contrast, the percentages of L1 in CA and RA were lower while that of CR was higher. As expected, CA was enriched with foundational gene pathways (ribosome, translation, and lipid transport), whereas RA was enriched with olfactory receptor (OR)‐related pathways (Figure 6B). No significantly enriched pathways (FDR < 0.01) were identified for CR.

We then spotted an ≈3 Mb genomic segment (chromosome 10: 126.16–129.28 Mb), harboring 19 housekeeping (HK) genes (mainly located at CA), 10 ORs (at RA), and 129 L1 repeats (at CR) (Figure 6C). The activation indexes of HK genes stabilized at high levels across the three cell types, while those of L1 repeats remained at low levels (Figure 6D). On the D^2^ plot, the housekeeping genes persistently enriched at the inner low‐density regions, while the L1 repeats enriched at the periphery (Figure 6E; Figure S19, Supporting Information), corresponding to their constitutive activation or repression, respectively.

ORs were a large gene family that expressed at mature OSNs for sensing odors.^[^ ^33^ ^]^ Correspondingly, their activation indexes increased during development (Figure 6D), and they moved on the D^2^ plot from the periphery (newborn OSNs), to inner high‐density (developing OSNs), and then toward lower density (mature OSNs), in consonance with their activation process (Figure 6E; Figure S18, Supporting Information). Additionally, for each individual OSN, only one specific OR expressed while all the others were repressed,^[^ ^33^ ^]^ which would leave featured distributions on D^2^ plot. Our D^2^ plot demonstrated that ORs collectively enriched inside nuclei with varying densities, corresponding to the active, intermediate, and repress and histone areas (Figure S20, Supporting Information), and reflecting their diverse levels of activation at the bulk level.

## 3 Conclusion

Inside the cell nuclei, the genome is compartmentalized into regions with different physical properties, to effectively execute their biological functions. Based on the reconstructed genome structures from single‐cell Hi‐C, we developed the D^2^ algorithm to define density and DisTP at a genome‐wide scale and integrated them into a two‐dimensional D^2^ plot. Different genetic and epigenetic markers exhibited distinct enrichment patterns on the D^2^ plot, indicating that these two physical properties, density and DisTP, were two pivotal parameters for identifying the functional compartmentalization of the genome structure. For instance, transcription‐related enzymes and active genomic sites altogether clustered at inner low‐density regions, whereas repressed heterochromatin markers enriched at inner high‐density or periphery regions.

In this study, we investigated the enrichments of architectural proteins (CTCF, YY1, and cohesin) on the D^2^ plot and revealed their largely overlapping enrichment zones, correlating to their cooperative functions. In addition, we spotted cohesin‐independent enrichments of YY1 and CTCF at inner low‐density regions, hinting their independent functions. Furthermore, all the architectural proteins located inside the nuclei, suggesting alternative mechanisms for genome construction at the nuclear periphery. L1 repeats, which were previously reported to promote heterochromatin compartmentalization,^[^ ^28^ ^]^ were found to mainly locate at the nucleic periphery, implying their possible involvement in maintaining genome construction.

In this study, we proposed a cross‐species transcriptional activation model on the D^2^ plot among different human and mouse cells. This model revealed that the distance to the periphery was negatively correlated with the transcriptional activities of genes, which seemingly contrasted to the center‐positioned nucleoli that were mainly composed of heterochromatin. The significantly low genome coverage (<5%) of nucleolus‐associated domains might render these regions neglected in our model.^[^ ^34^ ^]^ To examine the fitness of our model to reflect gene activation, mouse olfactory neurons at three differentiating stages were analyzed, and our model successfully identified the activated genes based on the change of density and DisTP. However, the underlying mechanism has not been explored. We assume that the translocation of the selected genes between distal regions with different density and DisTP is unfavorable, owing to the high cost of energy. Alternatively, the changes of local chromatin modifications, such as histone modification, assembling into distinct condensates through phase separation, and simultaneously altering surrounding density or DisTP, is feasible.

Notably, the D^2^ model of mouse cells, albeit being derived from mouse forebrain neurons, is capable of linking the structure with gene expression during OSN development, verifying its broad fitness and feasibility. Nonetheless, further verification and application of this model in other species is required, but these are currently unavailable owing to the lack of high‐quality single‐cell Hi‐C data. Our model would be applicable to most species, where sophisticated Mb‐scale spatial organization is essential for regulating gene expression. It might be helpful to elucidate human tumorigenesis analysis, which usually involves dramatic alteration of the genome structure.

Our study provides a new perspective for 3D genome analysis. Previous studies mainly focused on well‐known structures such as loops or TADs, which only revealed the spatial adjacency of focal structures. Contrarily, our D^2^ model allows for universal measurements of the genome structure and correlates the genomic structure with its function. Nonetheless, we speculate that physical properties of DNA act more as microenvironments to facilitate or repress gene transcription activity rather than direct and decisive factors, as protein structure to its function. In summary, our study paves the way for further methodological exploration to link genome structure with its function.

## 4 Experimental Section

### D^2^ for Detecting Density and DisTP

Python script D2.py implemented the D^2^ algorithm, which rapidly and accurately computed the DNA density and DisTP values based on the reconstructed genome structure. D^2^ algorithm can be found at https://github.com/xjtu‐omics/D2.

### Inputted Reconstructed Structures

Reconstructed genome structures, which were generated from single‐cell Hi‐C, were the main input for the D^2^ algorithm. Genome structures from two research groups were collected in this study (Table S1, Supporting Information). Stevens et al.^[^ ^16^ ^]^ processed single‐cell Hi‐C on eight individual haploid mouse ES cells and then reconstructed the nuclear structures, using the NucDynamics algorithm. The algorithm was designed based on molecular dynamic simulation. First, the genome was divided into 100 kb genomic bins, each of which represented a DNA particle in 3D structure. Then, a structure was constructed using the physical force field, including attraction, repel, and restriction. Using the simulated annealing algorithm, the structures were constructed to optimally simulate actual structures. The other group, Tan et al. developed a novel chromain conformation capture method, Dip‐C, which can detect more contacts than single‐cell Hi‐C. They omitted biotin pull down and conducted high‐coverage whole‐genome amplification (META). Then, they used a genome reconstruction algorithm, hickit, which was similar to NucDynamics and enabled the construction of diploid cells at a relatively higher resolution (20 kb).^[^ ^10^ ^]^

### Partition Particles by 3D Segmentation

Owing to the heterogeneity of nuclei sizes among cells, the reconstructed structures were not directly comparable among cells. This batch effect resulted in different nucleic radii and DNA densities. To address this, the DNA particles were placed inside a 3D mesh segmentation with equally shaped cubes, with cell‐specific cube length. The length was set as the average distance to the nearest *i*th particle of all particles from a given cell (*i* is the predetermined parameter, default as 7), to ensure the average number of DNA particles within a cube was approximately the same across cells. This predetermined parameter was not sensitive for further analysis, because the following analysis concentrated on the differences between the density and DisTP of genomic regions (e.g., lower‐density regions), while the change in this parameter increased or decreased density or DisTP universally. The segmentation was constructed according to the cube length and covered the particles. Then the spatial coordinate of particles was replaced using the cube coordinate. This normalization was based purely on DNA density, but the nucleic radius (half of the maximum distance among filled cubes) was adequately normalized (Figure S21, Supporting Information). Furthermore, this partition reduced the number of tasks from particles to cubes, thus efficiently speeding up and saving memory during calculation.

### Computing DNA Density

Once segmentation was performed, DNA density could simply be defined as the number of particles per cube. However, the arbitrary partition above could result in an inaccurate density. For example, if a DNA particle resided in the transiting regions between compacted and loose regions, it should be labeled as middle density. However, it may be partitioned into high‐density or low‐density cubes, instead of a middle one. To solve this problem, the density of a given cube was smoothened by its nearby cubes through the following equation

(1) $$D_{i}=\frac{\sum_{j\in S}C_{j}/∥\vec{i}-\vec{j}∥}{\mathrm{num}\left(S\right)}$$

where *D_i_* is smoothed DNA density of the spatial cube *i*, *C_i_* is the DNA particle number within the cube *i*, and *S* is the set of nearby cubes. The nearby range was set as 3. A longer range would be more optimized for calculation, but it was unnecessary as distant cubes contributed little.

### Computing Distance to Periphery

Previous methods for detecting DisTP were simple and, to a certain extent, inaccurate (Note S2, Supporting Information). One of them was developed in 2017 by Stevens et al.^[^ ^16^ ^]^ It first identified the empty cubes around the nonempty cubes as the surface. Then, they calculated the DisTP as the nearest distance to the surface. However, imaging experiments showed that chromocenters would repel DNA inside the nucleus,^[^ ^10^ ^]^ which would be wrongly defined as the nuclear surface in Steven's methods. Tan et al. used another metric, the distance to the nuclear center.^[^ ^10^ ^]^ This circumvented the chromocenter, but assumed the nuclei to be spherical. However, nuclei were always in different shapes. Therefore, a new methodology that accurately defined the periphery and then computed the DisTP was introduced.

In this method (Figure S1, Supporting Information), the outer empty cubes were first defined as the membrane cubes. They were started to define from the boundaries of cubes, and then were moved toward the inner cubes. The definition was stopped when filled cubes were encountered. To eliminate the dissociative outlier particles, the definition was stopped only when there were enough particles within a certain range (3 particles in 5 cubes by default). In other words, the faraway cubes with only one or two particles were defined as membrane cubes. Then, the DisTP for a given cube was calculated as the average value of the top 10 minimum distances to membrane cubes. The minimum distances were not used because they were discrete values and highly sensitive. For example, if one inner cube was incorrectly defined as a membrane cube, the cubes around it would be defined as periphery.

### D^2^ Plot: Construction of D^2^ Plot

To combine density and DisTP for further systematic analysis, a 2D density–DisTP matrix, the D^2^ plot was constructed. Outlier bins were eliminated during plotting. For the density axis, the distribution resembled the normal distribution. Therefore, the 1.96 * SD was selected from the center as the separating line, to retain 95% of the center bins. For the DisTP axis, all the bins were maintained near the periphery, but eliminated the top 5% bins further away from the periphery. The ranges were similar among the diploid cell types, even among the species (Table S4, Supporting Information). Therefore, a standard range was set for all diploid cells (1 to 3.2 for density, 1.21 to 16 for DisTP). Each axis was then divided into 15 equally shaped bins. The bin number was set arbitrarily, but it was showed that different bin numbers did not affect the enrichment patterns (Figure S22, Supporting Information). Each 2D bin at the D^2^ plot represented a specific physical state with a unique combination of density and DisTP. Notably, states with fewer than 200 particles were removed from subsequent analysis to ensure statistical significance (marked as empty).

### Computing Marker Enrichment Scores

To calculate the marker enrichment scores on the D^2^ plot, genetic data were weighted and averaged by the probability of the genomic segment appearing at the physical state, as shown below

(2) $$E_{a}=\frac{\sum_{G}P_{ga}\cdot NV_{g}}{\sum_{G}P_{ga}}$$

where *E_a_* is the computed enrichment score of the genomic marker in the physical state, *a*. *NV_g_* is the z‐scored normalized enrichment score of a genetic marker at the genome segment, *g*. *P_ga_* is the probability of the genome segment *g* appearing in the state *a*, defined as the ratio of the number of cells whose genomic segment *g* located at the state *a* with respect to the total cell number. Afterward, the enrichment scores were normalized by z‐score normalization.

Through this procedure, the density and DisTP of single cells were determined. There was another way of computing the enrichment scores, where the density and DisTP were averaged first

(3) $$E_{a}^{\prime }=\frac{\sum_{G_{a}}NV_{g}}{N\left(G_{a}\right)}$$

where $E_{a}^{\prime }$ is the alternative enrichment score derived from the average density and DisTP at physical state, *a*. *G_a_* is the collection of genomic segments whose average density and DisTP were located at state *a*. *N*(*G_a_*) is the number of collections. The average values delineated the bulk activities of a cell type more optimally, but were unable to represent the raw density and DisTP detected from the single cell. For example, the average values could not reflect the sparse distribution, for example, the multimodal distribution.

To test if the average density and DisTP would give similar enrichment patterns, the dip test was first carried out for unimodal distribution. All genomic bins were unimodal for both density and DisTP (Table S3, Supporting Information, percentage of both *p*‐value < 0.01: 98.11–99.88%). Then, the enrichment score analysis with average density and DisTP was repeated and it was found that it exhibited similar patterns to Figure 4 (Figure S23, Supporting Information), indicating that the average density and DisTP equivalently reflect biological activity.

### Stochasticity of Marker Enrichment Score

Considering the high cell stochasticity of density and DisTP, the stochasticity of marker enrichment scores was examined by computing their SD using the following equation

(4) $${\mathrm{SD}}_{a}=\sqrt{\frac{\sum_{G}P_{ga}\cdot {\left(NV_{g}-E_{a}\right)}^{2}}{\sum_{G}P_{ga}}}$$

where SD_*a*_ is the calculated SD of the marker enrichment score at physical state *a*. *NV_g_*, *P_ga_*, and *G* are defined as above. To compare SD with the mean enrichment scores, they were presented together at Figure S24 of the Supporting Information. The max–min normalization on mean values was utilized for comparison between markers. This normalization was also used on SD values. Therefore, markers with SD less than 0 denoted low stochasticity, while others with greater than 1 denoted high stochasticity. Certain markers (e.g., H3K27me3 and H3K9me3) showed lower SD than the mean value, indicating their consistent enrichment among cells. Nonetheless, most markers showed a comparable or larger SD, indicating high stochasticity. To test the robustness of the enrichment score analysis under such stochasticity, the analysis was repeated using only one cell (Figure S25, Supporting Information). The enrichment patterns, even from one cell, were highly similar to Figure 3, suggesting a variable despite the robust correlation between physical properties and genetic markers.

### Hierarchy Cluster and Property Strength

To obtain a transcriptional pattern on the D^2^ plot, 16 transcription‐related markers (Txn‐St, Txn‐W, Pr‐Ac, Pr‐W, En‐St, En‐W, H3K27ac, H3K36me3, H3K4me3, H3K4me2, Alu, H3K9mne3, H3K27me3, L1, Ht‐L; the abbreviations are spelled out in the legend of Figures S7 and S10, Supporting Information) were selected and the hierarchy cluster (scipy.cluster.hierarchy with parameter optimal_ordering = True, method = “ward”) was performed on physical states from the D^2^ plot. The hierarchy cluster number was set as 4 for both species.

The property strength reflected the intensity of impact of density or DisTP on the transcription level. The active markers were selected and the property strength was computed at a given DisTP as the slope between property changes (*x*) and marker enrichment score (*y*), as follows

(5) $$S_{d,p}=\mathrm{slope}\;\mathrm{of}\;\mathrm{linear}\;\mathrm{fitting}\;\left(x={\mathrm{MFG}}_{d,\;p},y=P_{d,p}\right)$$

where *S*_d,p_ is the computed property strength of property p at DisTP d. For density, the *x* input for linear fitting is the collection of all densities at a given DisTP and *y* is the corresponding marker enrichment score. For DisTP, *x* is the collection of the given DisTP and its nearby DisTP, and *y* is the corresponding enrichment score. The property strength reflected the association between the physical properties and the transcription level.

### Distribution of Lineage‐Specific Active Genes

The distribution of active genes was examined on the D^2^ plot, to validate the transcriptional activation model. Considering the nonuniform distribution of whole genome bins on the D^2^ plot, the enrichment scores of active genes over the background were computed. Specifically, the distribution of active genes was first calculated, and then it was divided by the distribution of all genomic bins as the background.

### Trajectories on D^2^ Plots

The activation index and its change were introduced to reveal the trajectories of a given genomic region on the D^2^ plot, which reflected its transcriptional activation modes (Figure S18, Supporting Information). Specifically, the activation index was defined as the sum of the enrichment scores of a given genomic bin on active or intermediate regions. Based on the activation index, the top 10% genomic bins were marked as the active bins, the bottom 10% as the repressed bins, and the others as medium. Three transcriptional modes: constantly active (CA), constantly repressed (CR), repressed to active (RA), were identified. During OSN maturation, the bins, which were active at all OSN cell stages, were marked as the CA bins. Conversely, the bins repressed at all stages were marked as CR bins. RA bins comprised bins that were not active at newborn OSNs, but active at mature OSNs.

The genomic positions of exons were obtained from the gff file, and those of L1s were obtained from RepeatMasker. Housekeeping genes were obtained from the HRT Altas v1.1 database.^[^ ^35^ ^]^ Their percentage of length at the given genomic bins was calculated.

The gene pathway enrichment analysis was performed by DAVID.^[^ ^36^ ^]^ Only KEGG pathways and GO terms were chosen for enrichment.

### Reference Genome

The mouse reference genome (GRCm38) and gene annotations (ALL) were downloaded from the GENCODE (https://www.gencodegenes.org/mouse/) M19 release.

The human reference genome (GRCh37) and gene annotations (ALL) were downloaded from the GENCODE (https://www.gencodegenes.org/human/) 39 release.

### Statistical Analysis

All of the Pearson correlations were two‐sided and were computed using scipy.stats.pearsonr. The linear fitting was conducted using scipy.stats.linregress, with parameter deg = 1. The sample size for each statistical analysis is available in the figure legends or in the Supporting Information. Fisher tests were two‐sided and computed by scipy.stats.fisher_exact.

### Preprocess of Genetic Markers

To integrate density and DisTP with other genomic data, the other data were transferred into the same indexed data. All data (except DamID‐LmnB1 data of mESC) were indexed by 20 kb genomic bins. For unphased data, the data were copied to two haplotypes.

### ChIP‐seq Data

ChIP‐seq sequenced protein binding sites. ChIP‐seq data of transcription factors (CTCF, YY1, RAD21, SMC3, POLR2A, POLR2Ap5 for human GM12878 cells) and histone modifications (H3K4me2, H3K4me3, H3K9me3, H3K27ac, H3K27me3, H3K36me3 for both human GM12878 cells and mouse neonatal neurons) were collected. These data, with different types of processed files, were collected from ENCODE. Here the fold change was utilized over the control file, because it provides whole genome protein binding information. This file was formatted as BigWig and was transformed to a bedgraph file using the UCSC script bigWigToBedGraph. Then bedtools intersect were used to find the intersected regions between the index file and the marker bedgraph file. Next, the fold change of all 20 kb bins was weight‐averaged and they were indexed according to the index file.

### Genomic Repeat

The repeat data were obtained from RepeatMasker. Owing to the low resolution of the 3D genome, only broadly distributed repeats would be statistically significant for enrichment. Therefore, repeats with less occurrences were filtered out. Only repeats with more than 20 000 occurrences were left. The remaining repeats with the index file were intersected and then their percentage at a given genomic bin was calculated, and finally they were indexed in the aforementioned procedure.

### Chromatin State

Chromatin states described the transcriptional states of genomic loci. They were calculated using HMM and based on chip‐seq data.^[^ ^37^, ^38^ ^]^ Because chromatin states were discrete states, intersections, calculated percentages, and indexed were performed through processes similar to those of the repeats. Each genomic bin had a value for each chromatin state, representing the length of the state within that bin.

### CpG

The CpG data were downloaded from ENCODE, formatted as bedMethyl. Sites were filtered out without reads. The remaining sites were intersected with the index file, the median CpG percentages were calculated in a given bin, and finally they were indexed using the aforementioned procedure.

### DamID‐Lmn for Detecting LADs

DamID‐seq identified protein binding sites by expressing the proposed DNA‐binding protein as a fusion protein with DNA methyltransferase. Using DamID‐seq to detect the binding sites of Lmn‐B1 (Lamin‐B1), the main component of nuclear lamina, is a popular method of detecting LADs. The mESC LAD experimental data were utilized.^[^ ^39^ ^]^ Because these data were discrete like genomic repeats, the data were intersected, calculated, and indexed in the same way as the repeats. Notably, the bin size was 100 kb for the used mES cells.

To validate DisTP, experimental LAD data were compared to the probability of appearing at the nuclear periphery (percentages of cells whose given genomic bin had DisTP below 2). As they were both indexed by the same index file, they were associated with their index. The outliers were eliminated using three‐sigma limits.

Noteworthy, due to the small number of available cells (*n* = 8), the possible values for simulated LAD data are too sparse at 100 kb resolution. Therefore, the nearby bins were concatenated to 2 Mb resolution, in order to provide continuous percentages. The number of 2 Mb genomic bins is 1103. The two‐sided Pearson correlation was computed using scipy.stats.pearsonr.

### Stochasticity of Density and DisTP

To explore the stochasticity of DisTP, its standard deviations were computed (SDs; Figure S4, Supporting Information). SD was computed among cells of given genomic bins, thereby reflecting the cell heterogeneity. The lower SD meant a more stable DisTP value among cells.

The stochasticity of density was computed similarly to DisTP. In contrast with DisTP, the lower SD regions of density showed obvious cell‐type specificity. Therefore, the Pearson correlation between density mean and density SD (scipy.stats.pearsonr) was conducted, to identify the values of density that were more stable.

## Conflict of Interest

The authors declare no conflict of interest.

## Authors Contribution

Y.C. developed the D^2^ algorithm with the help of X.Y. Y.C. downloaded, analyzed, and interpreted the data in current studies. P.J. and T.W. provided help for analyzing data. Y.C. was the major contributor in writing the manuscript. T.G. helped with the benchmark. X.Y., D.X., and K.Y. revised the manuscript. K.Y. supervised the whole program. All authors read and approved the final manuscript.

## Supporting information

Supporting Information

## Data Availability

The reconstructed genome structures analyzed during the current study are available in the GEO repository: GSE117876, GSE94489, GSE121791 and GSE162511. The ChIP‐seq data analyzed during the human GM12878 study are available in the ENCODE repository: ENCFF364OXN (CTCF), ENCFF368HBX (POLR2A), ENCFF002UPS (POLR2Ap5), ENCFF631VCL (YY1), ENCFF235BXX (SMC3), ENCFF567EGK (RAD21), ENCFF828CQV (H3K4me2), ENCFF674QZB (H3K4me3), ENCFF776OVW (H3K9me3), ENCFF180LKW (H3K27ac), ENCFF594HSG (H3K27me3), ENCFF662QFK (H3K36me3) and ENCFF279HCL (CpG). The ChIP‐seq data analyzed during the mouse forebrain tissue study are available in the ENCODE repository: ENCFF879HIK (H3K4me2), ENCFF050AHA (H3K4me3), ENCFF965IFF (H3K9me3), ENCFF422JMO (H3K27ac), ENCFF742UNH (H3K27me3), ENCFF213BDE (H3K36me3). The chromatin states data of human GM12878 are available in GEO GSE26386, while that of mouse forebrain tissue are available at UCSC Table Browser. The Dam‐ID‐seq data of Lmn‐B1 of mESC are available at GEO GSE123586. Code for D2 algorithm is available at https://github.com/xjtu-omics/D2.
