# Supplementary material for: D2 Plot, a Matrix of DNA Density and Distance to Periphery, Reveals Functional Genome Regions
Source: Adv Sci (Weinh). 2022 Aug 30;9(30):2202149. doi: 10.1002/advs.202202149 (PMC9596860; doi:10.1002/advs.202202149)
Supplement: Supplementary file 1 — Supporting Information [file ADVS-9-2202149-s001.pdf]

Supporting Information

D^2 plot, a matrix of DNA density and distance to periphery, reveals functional genome regions

*Yizhuo Che, Xiaofei Yang, Peng Jia, Tingjie Wang, Dan Xu, Kai Ye**

Corresponding Author. Email:kaiye@xjtu.edu.cn (K.Y.)

This PDF file includes:

Supplementary Note 1 to 5

Tables S1 to S5

Figures S1 to S25

Supplementary Note 1. D^2 is robust against different genome reconstruction methods

To test the robustness of D^2 against different reconstruction methods, we applied several methods to the same cells and compared their outcomes from D^2. The tested methods were shown in **Table R1**. Eight mES cells were used as test dataset, because 1) genome structures of these eight cells are available for several methods (SCL, Si-C); 2) genome of other diploid cells is too large to reconstruct for some algorithms. 100kb resolution is used for all of the tested methods.

Table R1. Different reconstruction methods for single-cell Hi-C

Method	Ref.	Sampling algorithm	WG? [a)]	State
**NucDynamics**	Stevens et al., 2017	Molecular dynamics	√	Finished
**Dip-C/hickit**	Tan et al., 2018	Molecular dynamics	√	Finished
**ShRec3D**	Lesne et al., 2014	Short distance + MDS	×	Code cannot be used [b)]
**MBO**	Paulsen et al., 2015	Short distance + MDS	×	Code unavailable [c)]
**SCL**	Zhu & Wang, 2019	Lattice based simulation	×	ChrX finished
**Si-C**	Meng et al., 2021	Bayesian theory	√	Finished

[a)] WG is short for whole-genome. If √, this algorithm can generate whole-genome structure. If ×, it can't. [b)] Code for ShRec3D is not adopted for single-cell Hi-C data, though the algorithm is theoretically feasible. [c)] Link to the code for MBO is lost.

Three (NucDynamics, Dip-C and Si-C) of the four available methods were able to reconstruct whole-genome structure. By comparing the average values of DNA density and DisTP (both the output of D^2), we revealed high correlations between these methods (Pearson correlation coefficient (PCC): 64.1%-94.96%, **Figure R1**), indicating that D^2 was robust against different reconstruction methods.

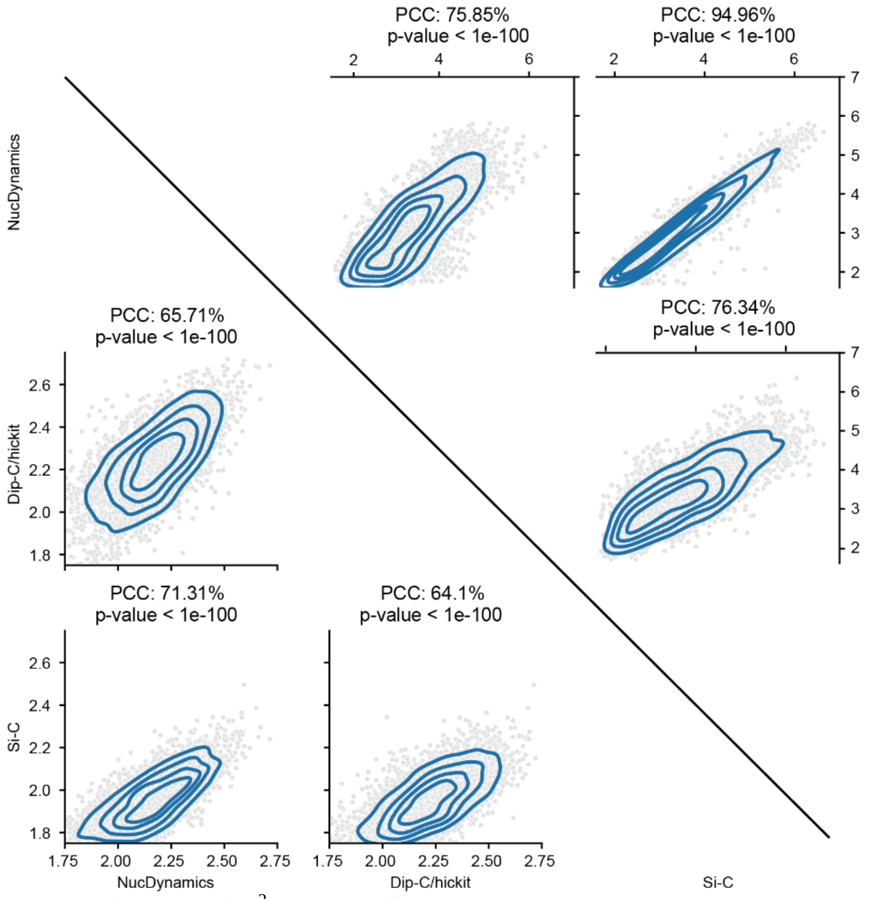

Figure R1. Comparison of output of D^2 among different reconstruction methods. The lower three sub-figures denote the correlation of DNA density, while the upper three with DisTP. The reconstruction of Dip-C is re-implemented and renamed as hickit. PCC is short for Pearson correlation coefficient. Pearson correlation is conducted by scipy.stats.pearsonr.

SCL can only reconstruct single-chromosome structures. Therefore, we reconstructed the structures of chromosome X with different methods, to test the robustness of D^2 (**Figure R2**). Although PCCs between SCL and other methods showed medium correlation (31.46%-59.24%), the overall correlations (52.66%-74.91% for PCCs not including SCL) revealed the outputs of D^2 were correlated among different methods.

In summary, we tested the outcomes (DNA density and DisTP) of D^2 among different reconstruction methods, and the high correlations imply that D^2 is robust against different methods.

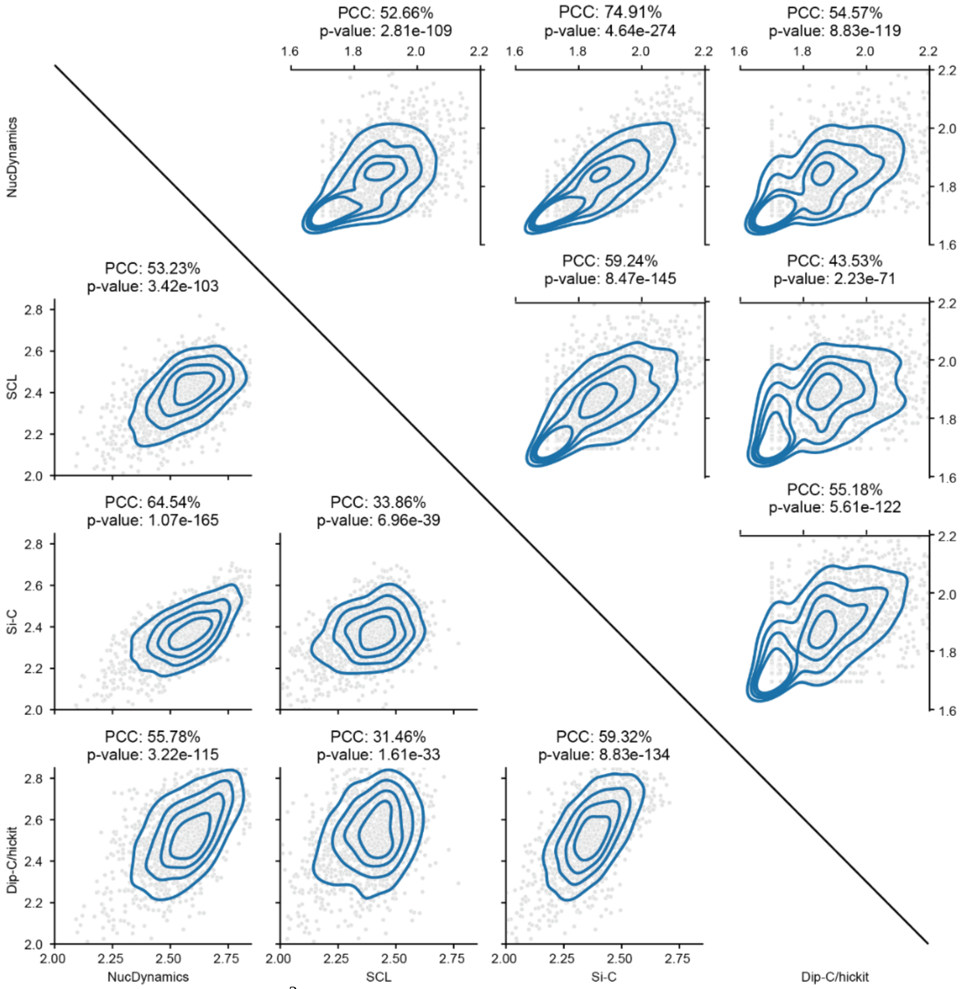

Figure R2. Comparison of output of D^2 among different reconstruction methods, using only chromosome X. The lower six sub-figures denote the correlation of DNA density, while the upper six with DisTP. The reconstruction of Dip-C is later re-implemented and renamed as hickit. PCC is short for Pearson correlation coefficient. Pearson correlation is conducted by scipy.stats.pearsonr.

Supplementary Note 2. Assessment of different methods for DisTP detection.

Two previous methods detected DisTP from 3D models of genome structures. The first is named as NucDynamics, by Stevens et al.[1] It firstly defined the empty cubes around the nonempty cubes as surface. Then, they calculated the DisTP as the nearest distance to the surface. However, the chromocenters which repelled DNA inside the nucleus, which would be wrongly defined as nuclear surface in Steven's methods. We tested their methods in GM12878 cells, and the example cell was shown in **Figure R3**. It is clear that the genomic bins around the chromocenters are wrongly assigned as periphery.

Tan et al. used another metrics, the distance to nuclear center. This avoided to deal with the chromocenter, but it assumed that the nuclei were sphere. However, nuclei were always in different shapes. We tested it again on GM12878 cells. The DisTP of bins near the nuclear periphery are sometimes wrongly computed (**middle bottom of Figure R3**).

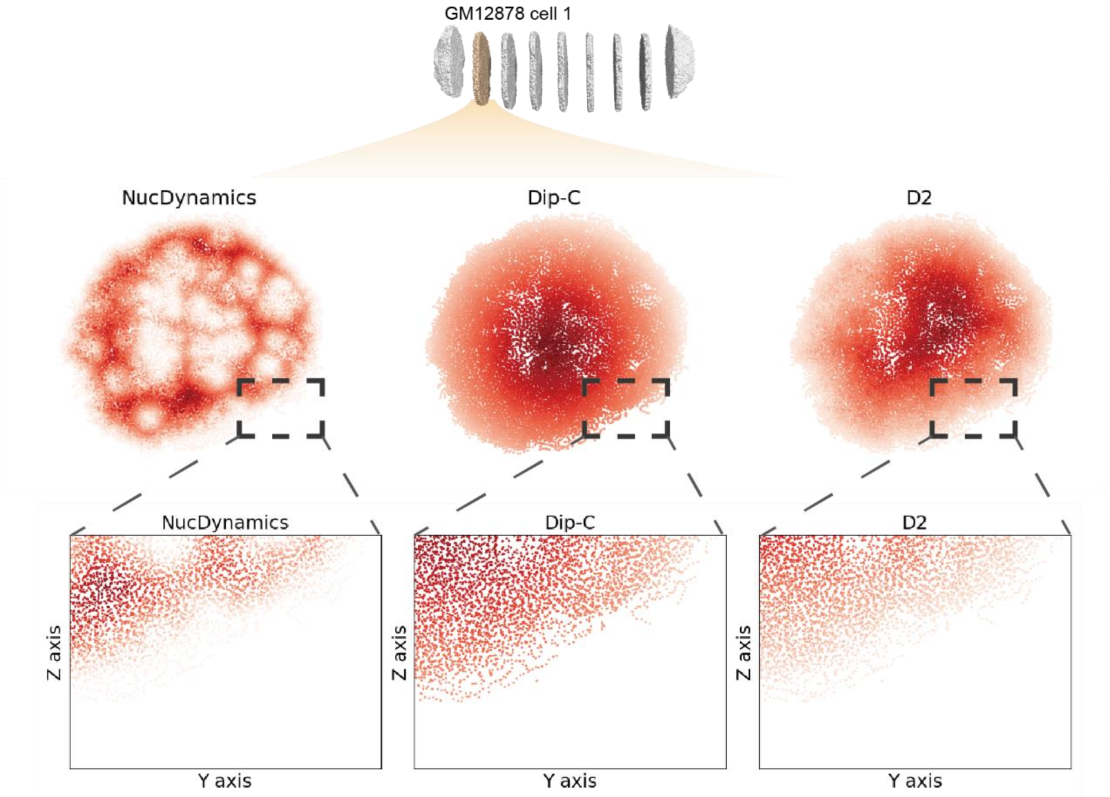

Figure R3. DisTP detection of different methods on exampled GM12878 cell. The color of each dot denotes the DisTP value. The darker the color, the higher DisTP is. We only showed one slice along the x axis of exampled cell (GM12878 cell 1). The x coordinate is in the range of 20% to 28% of whole x range. The sections of the slice were shown at the bottom, to zoom into the nuclear periphery.

Next, we compared the enrichments of histone modifications from different DisTP detection methods. Firstly, we computed the enrichments of active mark, H3K36me3 (**Figure R4**). D^2 and Dip-C showed similar results that H3K36me3 enriched at nuclear inside.

NucDynamics, however, failed to reveal this enrichment, owing to their inaccurate detection around the chromocenters.

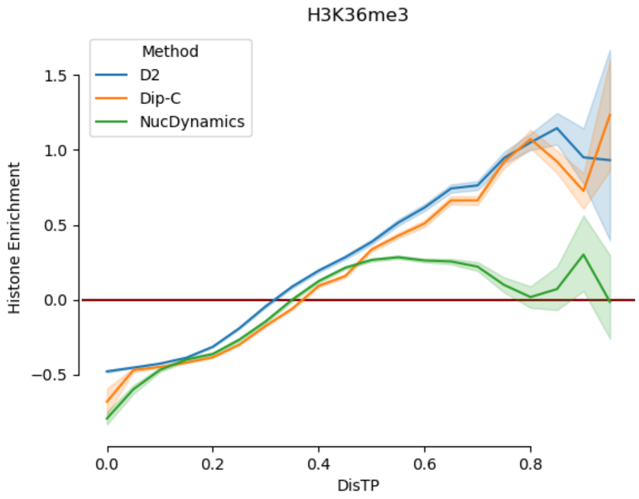

Figure R4. Line plot of enrichment of H3K36me3 at different DisTP, with different DisTP detection methods.

Supplementary Note 3. Assessment of DisTP detection using inverted genome structures.

In order to reveal the correlative nature between single-cell Hi-C signals and computed DisTP, we applied D^2 algorithm to the adult rods, the nuclear structures of which are inverted.[7] Specifically, the inner euchromatin and periphery heterochromatin exchanged their position. Therefore, we argued such dataset is helpful for DisTP validation.

The genome structures of adult rods are captured by Dip-C and reconstructed by hickit [8]. We applied D^2 to these structures to compute DNA density and DisTP. We plotted the enrichment patterns of genomic repeats on D^2 plot (Alu and L1 shown here, **Figure R5A**). These patterns are completely different from "conventional" cells. Alu, which normally enriched at inner nuclei, resided away from nuclear center but still preferred low-density regions. L1, on the other hand, moved from nuclear periphery to the nuclear center, hinting the inversion of the nuclear organization. The correlations of DisTP from different cell types were shown in **Figure 2B**. The correlations between rods and other conventional cell types are slightly negative (-14.91% to -29.99%), while the correlations among conventional cells are significantly positive (74.56% to 87.33%). The correlations indicated that adult rods adopted different mechanism of nuclear organization compared to other cell types. In summary, D^2 is able to detect DisTP for both conventional and inverted nuclei.

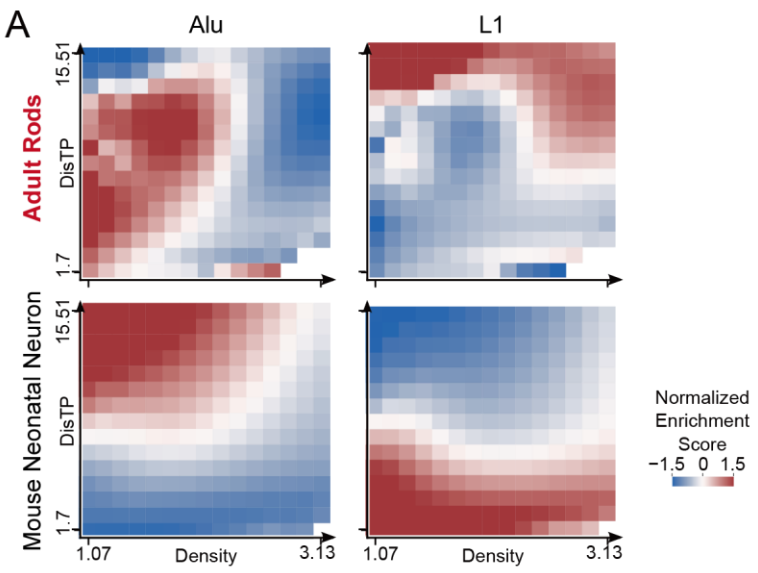

Figure R5. D^2 is capable of discovering the inverted nuclear structures of adult rods. The enrichments of genomic repeats (Alu and L1) on D^2 plot of adult rods (inverted genome structure) and neonatal neurons (conventional genome structure). The enrichment scores for the bottom right physical states were not shown owing to its small number (n<200) of genomic segments.

Supplementary Note 4. Define the transcription levels for whole chromosome 1

We extended our analysis on gene expression prediction to divide the genome into groups with different patterns of activation indexes. However, due to the memory limit, only bins from chromosome 1 (maternal) were analyzed. We first selected out the genomic bins with low activation indexes among all 12 cell types. 4254 out of 9516 (44.7%) bins were found and named as the constantly repressed (CR, blue) cluster (**Figure R6A**). CR significantly enriched for L1 repeats (Chi-square test p-value: 1.5×10^{-45}), compared to background (whole chromosome 1) (**Figure R6C**). The genes located at CR expressed at low level, compared to background (**Figure R6D**).

The rest of genomic bins were classified by hierarchy cluster based on the activation indexes (**Figure R6A**). Some of the clusters exhibited high activation indexes among the majority of cell types, for example the constantly active (CA, red) cluster. As expected, CA enriched for housekeeping genes (Chi-square test p-value: 1.3×10^{-62}), and expressed at higher levels compared to background (**Figure R6C and R6D**), indicating the strong transcription activity among all cell types.

Other clusters presented cell-type specificity. For example, the activation indexes of tissue specific (TA, yellow) cluster were only high at Cortical L6 cells, while the average indexes were below zero in others. The corresponding lineage-specific genes significantly enriched at TA (Chi-square test p-value: 8.0×10^{-19}).

In summary, activation index, which is generated from D^2 plot, shows a positive correlation with gene expression and is capable of dividing the genome into transcriptional active and repressed regions.

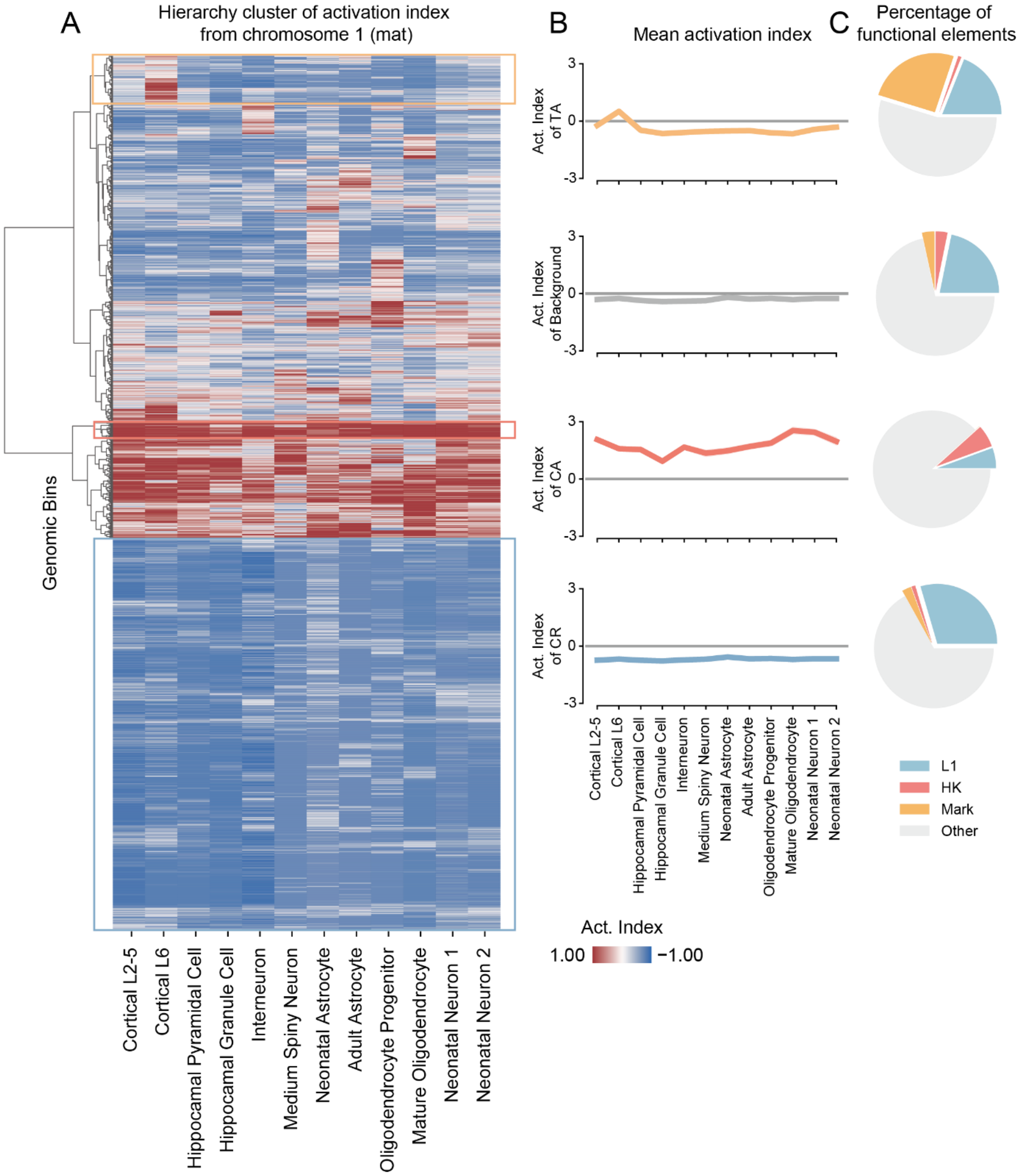

Figure R6. Genomic clustering of chromosome 1 (maternal) by activation indexes. **(A)** Hierarchy clustering of genomic bins (n=9516). The CR cluster (blue) is selected out first. Then the rest bins are clustered by scipy.cluster.hierarchy with parameter method=”ward”. The CA and TA clusters are found by scipy.cluster.hierarchy.fclust with the number of clusters set as 7. **(B)** Line plots of mean activation index. The horizontal gray line marks zero, for that activation indexes are Z-normalized. **(C)** Pie charts of functional elements on each cluster. Only L1 repeats, lineage-specific genes of cortical layer cells (Mark) and housekeeping genes (HK) are calculated. We used the whole chromosome at the background in (B) and (C).

Supplementary Note 5. Complex patterns on H3K9me3 enrichments on D^2 plot.

In order to explain the complex patterns of H3K9me3, we firstly manually draw the expected patterns (**Figure R7A**). Basically, H3K9me3 is expected to locate at nuclear periphery, since it enriched at LADs.[9] The chromocenters in mouse nuclei also enrich for this mark, therefore high enrichments on inside high-density regions are also expected in mouse. However, this enrichment should be missing in human nuclei. Strong repels are also anticipated in inside low-density regions for both human and mouse nuclei, since these regions are filled with active euchromatin.

By comparing to the real enrichments on D^2 plots, the main differences located at the nuclear periphery. This differences probably result from the short sequencing length (36 nt) for H3K9me3 ChIP-seq data, which is difficult to map to the periphery regions that are filled with long repeats (such as L1 repeats, shown in Figure 3D). Nonetheless, the longer sequencing length (for example 250 nt in Dip-C) is able to map to these regions. In summary, we could detect valid DNA density and DisTP for these regions, but failed to detect the H3K9me3 enrichments owing to the short sequencing length, therefore resulting in these complex patterns.

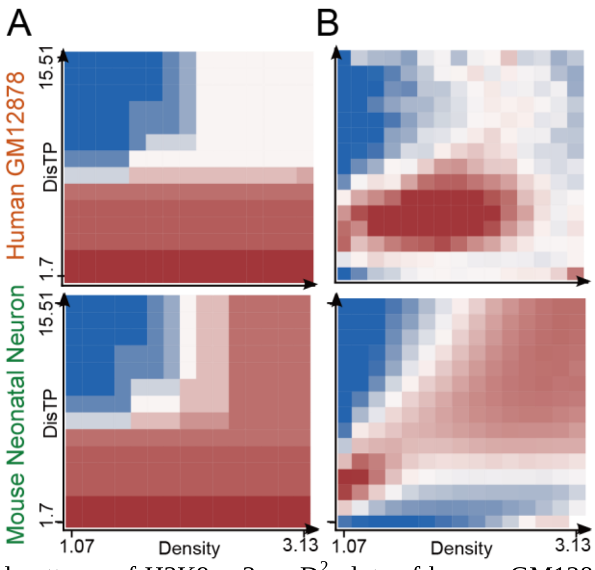

Figure R7. Expected and real patterns of H3K9me3 on D^2 plots of human GM12878 cells and mouse neonatal neurons. **(A)** Expected patterns of H3K9me3. We manually created the enrichment patterns according to the previous knowledges about the enrichments of H3K9me3. **(B)** Real enrichment patterns of H3K9me3.

Table S1. Statistical table for reconstructed genome structures

Species	Tissue	Source	Method	Cell Type	N [a)]	Ploidy
Human	Blood	Tan et al., 2018 [2]	E [b)]: Dip-C<br>R: Dip-C	GM12878	16	Dip. [d)]
				PBMC [c)]	18	
Mouse	NA	Nagano et al., 2017 [1]	E: Single-cell Hi-C<br>R: NucDynamics	Embryonic stem cell	8	Hap. [e)]
Mouse	Retina	Tan et al., 2019 [8]	E: Dip-C<br>R: Dip-C	Adult Rods	56	Dip.
Mouse	Main olfactory epithelium	Tan et al., 2019 [8]	E: Dip-C<br>R: Dip-C	Newborn OSN [f)]	60	Dip.
				Developing OSN	74	
				Mature OSN	143	
Mouse	Cortex and Hippocam-pus	Tan et al., 2021 [10]	E: Dip-C<br>R: Dip-C	Cortical L2–5 Pyramidal Cell	94	Dip.
				Cortical L6 Pyramidal Cell	33	
				Hippocampal Granule Cell	86	
				Hippocampal Pyramidal Cell	74	
				Interneuron	57	
				Mature Oligodendrocyte	41	
				Medium Spiny Neuron	19	
				Microglia Etc.	4	
				Neonatal Astrocyte	27	
				Adult Astrocyte	19	
				Oligodendrocyte Progenitor	20	
				Neonatal Neuron 1	237	
				Neonatal Neuron 2	110	

[a)] N is short for number of cells. [b)] E is short for experimental method, while R is short for reconstructed algorithm. [c)] Dip. is short for diploid. [d)] PBMC is short for peripheral blood mononuclear cell. [e)]Hap. is short for haploid. [f)] OSN is short for olfactory sensory neuron.

Table S2. Correlations between density and DisTP

Cell Type	Fitting Slope	R-square	PCC [a)]	*p*-value [b)]	Sample Size [c)]
GM12878	-0.06	0.0	-0.65%	2.39e-41	4.2e6
PBMC	-0.89	0.01	-9.44%	0.0	4.9e6
Newborn OSN	5.63	0.43	65.82%	0.0	1.4e7
Developing OSN	6.30	0.47	68.57%	0.0	1.7e7
Mature OSN	5.29	0.33	57.38%	0.0	3.4e7
Cortical L2-5 Pyramidal Cell	4.06	0.21	45.92%	0.0	2.2e7
Cortical L6 Pyramidal Cell	3.20	0.14	37.94%	0.0	7.7e6
Hippocampal Pyramidal Cell	3.76	0.18	42.81%	0.0	1.7e7
Hippocampal Granule Cell	3.66	0.17	41.55%	0.0	2.0e7
Interneuron	3.79	0.18	42.98%	0.0	1.3e7
Medium Spine Neuron	3.97	0.25	49.80%	0.0	4.4e6
Neonatal Astrocyte	4.33	0.29	54.17%	0.0	6.3e6
Adult Astrocyte	2.44	0.09	29.39%	0.0	4.4e6
Oligodendrocyte Progenitor	3.71	0.21	46.19%	0.0	4.7e6
Mature Oligodendrocyte	2.72	0.10	31.71%	0.0	9.6e6
Neonatal Neuron 1	5.34	0.33	57.17%	0.0	5.5e7
Neonatal Neuron 2	4.86	0.33	57.85%	0.0	2.5e7

[a)] PCC is short for Pearson correlation coefficient. [b)] p-value is computed by Pearson correlation. [c)] Sample size denoted the number of genomic segments used in Pearson correlation.

Table S3. Means and SDs of density and DisTP

Cell Type	Den. [a] Mean	Den. SD [b]	DisTP Mean	DisTP SD
GM12878	2.260	1.465	6.499	4.547
PBMC	2.323	1.457	6.601	4.373
Newborn OSN [c]	2.179	1.516	7.275	4.830
Developing OSN	2.200	1.526	7.438	4.563
Mature OSN	2.193	1.552	7.185	5.221
Mature Oligodendrocyte	2.145	1.509	6.565	4.631
Neonatal Neuron 1	2.169	1.510	7.331	4.716
Neonatal Neuron 2	2.162	1.528	7.004	4.832
Cortical L2-5 Pyramidal Cell	2.079	1.512	6.555	5.009
Cortical L6 Pyramidal Cell	2.077	1.509	6.416	4.851
Hippocampal Pyramidal Cell	2.103	1.505	6.578	4.732
Hippocampal Granule Cell	2.092	1.509	6.526	4.837
Interneuron	2.075	1.515	6.439	5.039
Medium Spiny Neuron	2.062	1.513	6.515	4.723
Neonatal Astrocyte	2.168	1.522	6.880	4.657
Adult Astrocyte	2.126	1.504	6.421	4.463
Oligodendrocyte Progenitor	2.126	1.514	6.618	4.693

[a] Den. is short for density. [b] SD is short for standard deviation. [c] OSN is short for olfactory sensory neurons.

Table S4. The density and DisTP boundaries of D^2 plot

Cell Type	DisTP Min	DisTP Max	Den.[a)] Min	Den. Max
mESC	1.21	6.70	1.01	3.10
GM12878	1.21	16.01	1.32	3.16
Newborn OSN [b)]	1.21	18.49	1.12	3.34
Developing OSN	1.21	18.15	1.08	3.43
Mature OSN	1.21	18.31	1.11	3.33
Cortical L2-5 Pyramidal Cells	1.21	16.34	1.07	3.14
Cortical L6 Pyramidal Cells	1.21	15.63	1.06	3.12
Hippocampal Pyramidal Cell	1.21	16.34	1.08	3.16
Hippocampal Granule Cell	1.21	16.59	1.06	3.17
Interneuron	1.21	16.08	1.07	3.11
Medium Spine Neuron	1.21	16.54	0.95	3.28
Neonatal Astrocyte	1.21	16.27	1.06	3.29
Adult Astrocyte	1.21	15.15	1.11	3.12
Oligodendrocyte Progenitor	1.21	15.81	1.05	3.23
Mature Oligodendrocyte	1.21	15.71	1.12	3.14
Neonatal Neuron 1	1.21	17.87	1.13	3.25
Neonatal Neuron 2	1.21	17.00	1.07	3.31

[a)] Den. is short for density. [b)] OSN is short for olfactory sensory neurons.

Table S5. Dip test for density and DisTP

Cell Type	Both Sig. [a]	Den. [b] Sig. Not DisTP	DisTP Sig. Not Den.	Both Not Sig.
Newborn OSN [c]	0.00%	0.08%	0.02%	99.88%
Developing OSN	0.00%	0.06%	0.05%	99.88%
Mature OSN	0.00%	0.17%	0.33%	99.48%
Cortical L2-5 Pyramidal Cell	0.01%	0.08%	0.45%	99.44%
Hippocampal Pyramidal Cell	0.02%	0.05%	0.21%	99.70%
Hippocampal Granule Cell	0.03%	0.08%	0.94%	98.93%
Interneuron	0.00%	0.05%	0.25%	99.68%
Neonatal Neuron 1	0.04%	1.47%	0.36%	98.11%
Neonatal Neuron 2	0.02%	0.11%	0.18%	99.69%

Dip test testifies if the distribution is multimodal. For statistical significance, only cell types with cell number larger than 50 are examined. Density and DisTP are examined separately. The significant bins (p-value < 0.05) suggested that it obeyed multi-modal distribution, indicating that the density or DisTP values of different cells could be divided into sub-groups. [a] Sig. is short for significant (p-value < 0.01). If significant, the distribution is believed to be multimodal. [b] Den. is short for density. [c] OSN is short for olfactory sensory neurons.

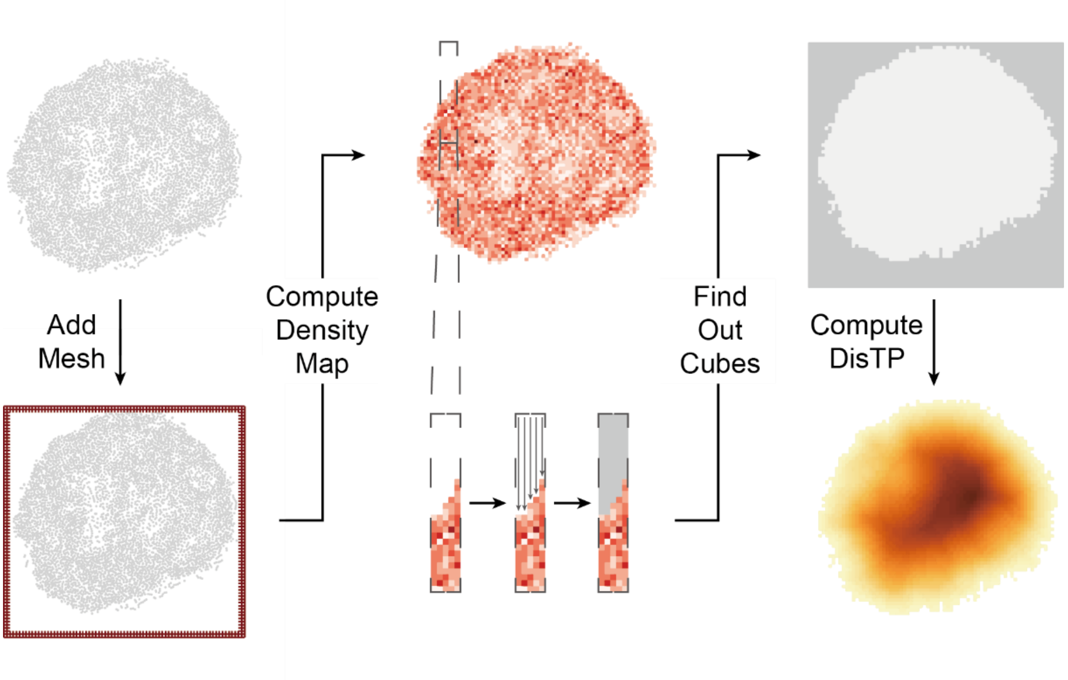

Figure S1. Illustration for DisTP detection method.
A section of an example cell (GM12878 cell 01) was used for this illustration. We only plotted the edges of the mesh instead of the whole one in "mesh" subplot (bottom left) for clear visualization. The mesh covers all over the structure. We showed a part of the structure in the bottom middle plots, in order to visualize the definition of out cubes. The colors in DisTP plots are same as Figure 1B.

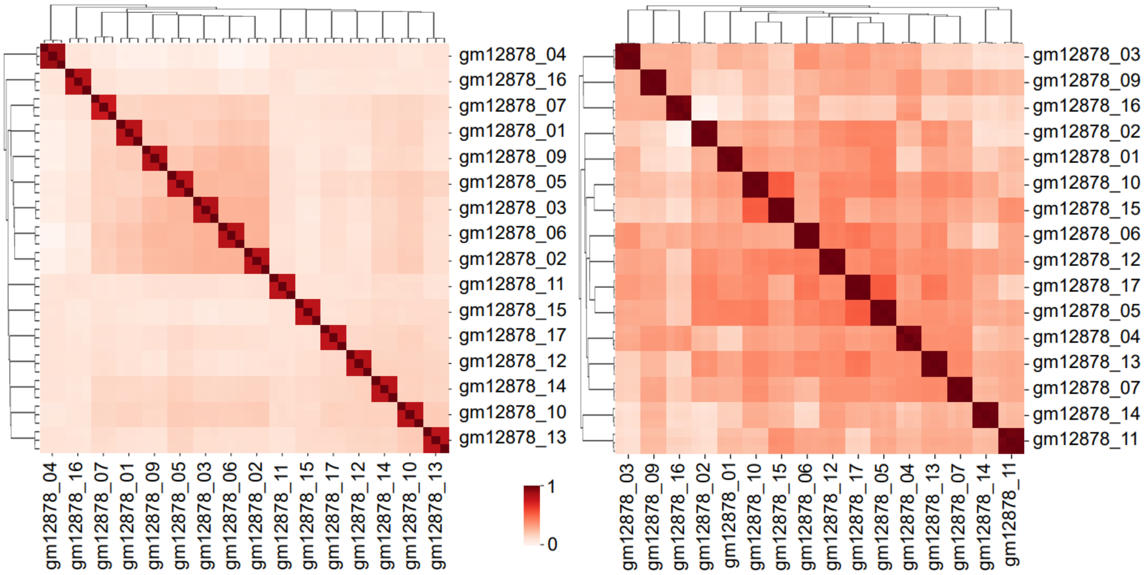

Figure S2. The hierarchy cluster of similarity matrix between different replicates. Replicates from one cell were clustered together, shown as the clustered panels consisting of three replicates. For clear visualization, the clustered three replicates of one cell were labeled together with its cell name.

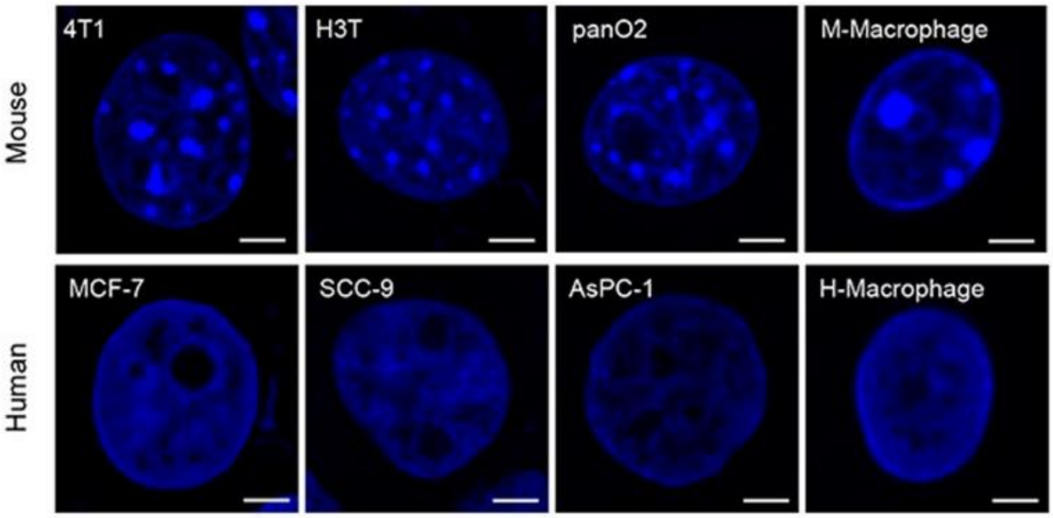

Figure S3. Nuclei images stained by DAPI.
These images were adopted from Wang et al.[11] Since DAPI binds to DNA unbiasedly, its intensity reflects DNA density. In mouse cells, deep-stained chromocenters appeared at the nuclear interior.

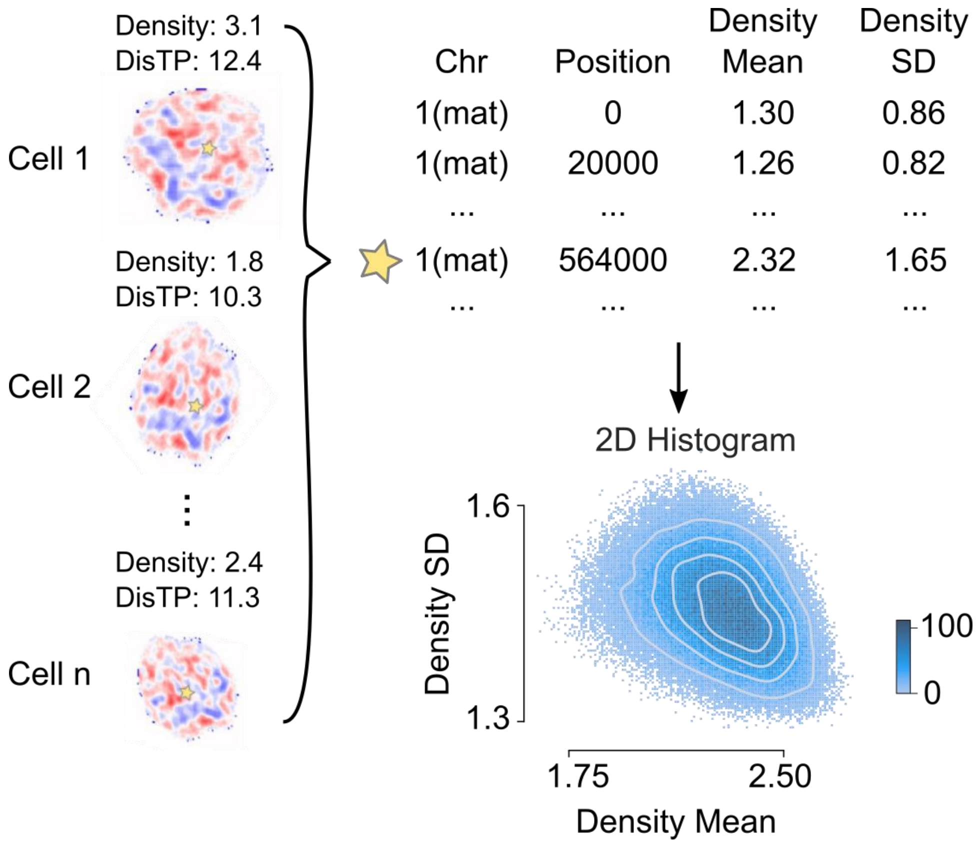

Figure S4. Illustration of computing SDs of density.
The left three sub-panels showed the density and DisTP value of the given genomic segment. These sub-panels were the nuclei sections colored by density (red for high density while blue for low). The right sub-panels indicated that Figure 2C and S5 were drawn by whole genomic bins. See Supplementary Methods for details.

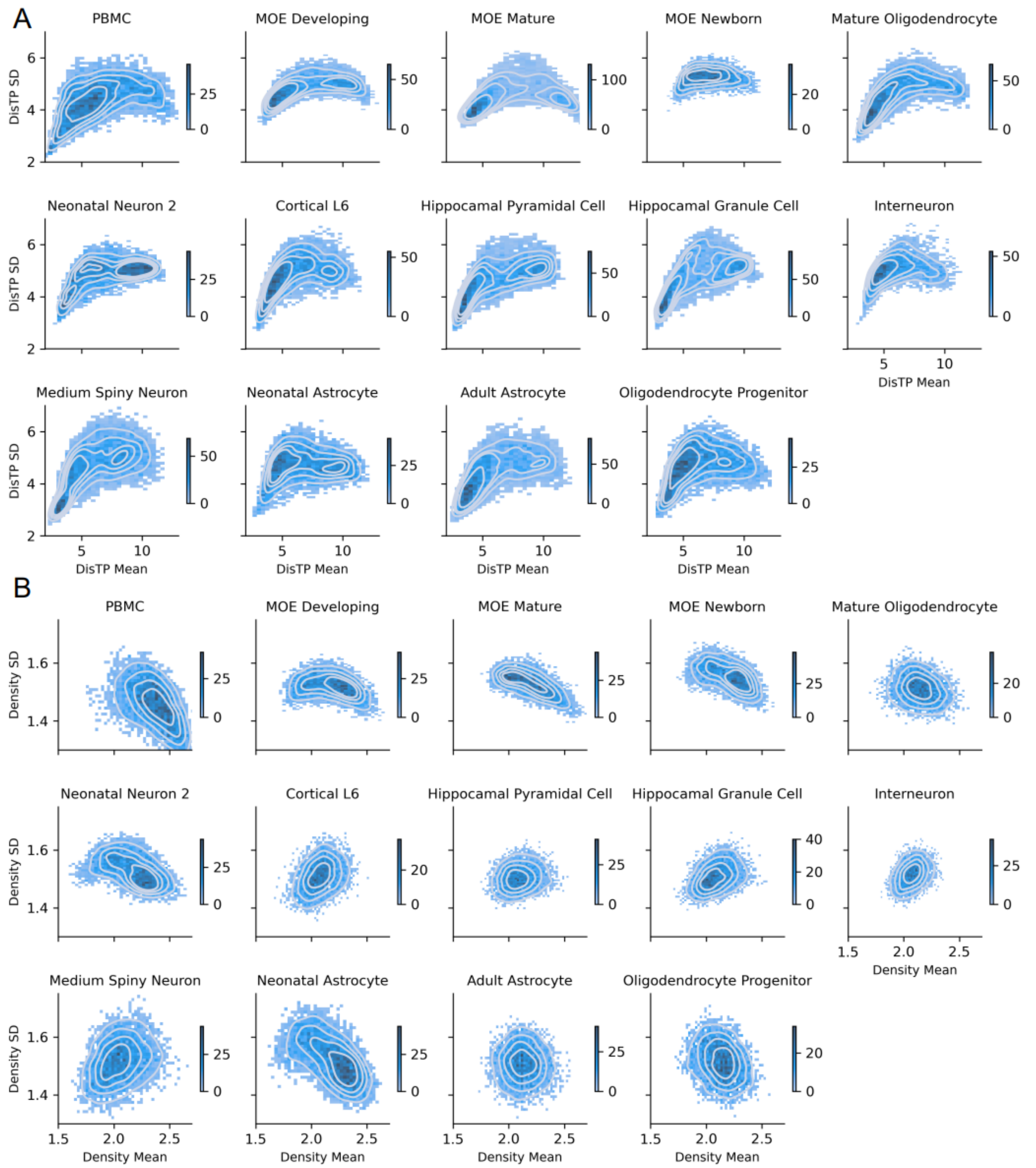

Figure S5. 2D histogram and KDE plots of mean and SD for additional 14 cell types. The other three cell types could be found at Figure 2C. (A) showed the DisTP mean vs. DisTP SD while (B) showed density.

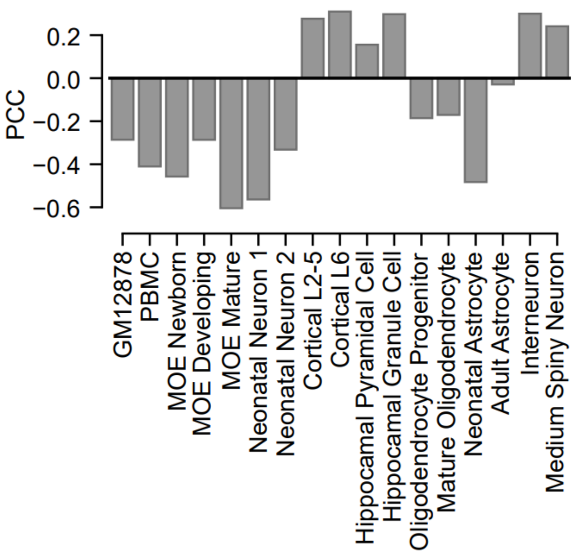

Figure S6. PCC between density mean and density SD of different cell types.
Six mature neuron cells exhibited positive correlation while the other cell lines showed negative correlation. Sample size for each cell type is same as Table S2.

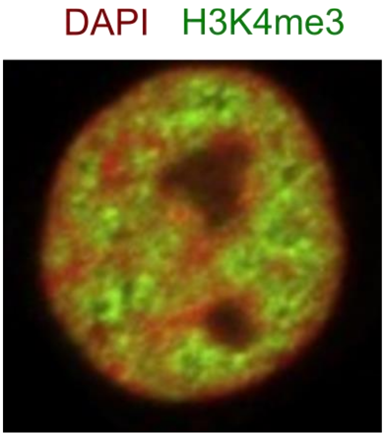

Figure S7. Human fibroblast nuclei stained with DAPI (red) and H3K4me3 (green), from Solovei et al.[12]

Here we could observe that H3K4me3 located at DAPI-weak (low-red signal) regions, suggesting that H3K4me3 located at low-density regions.

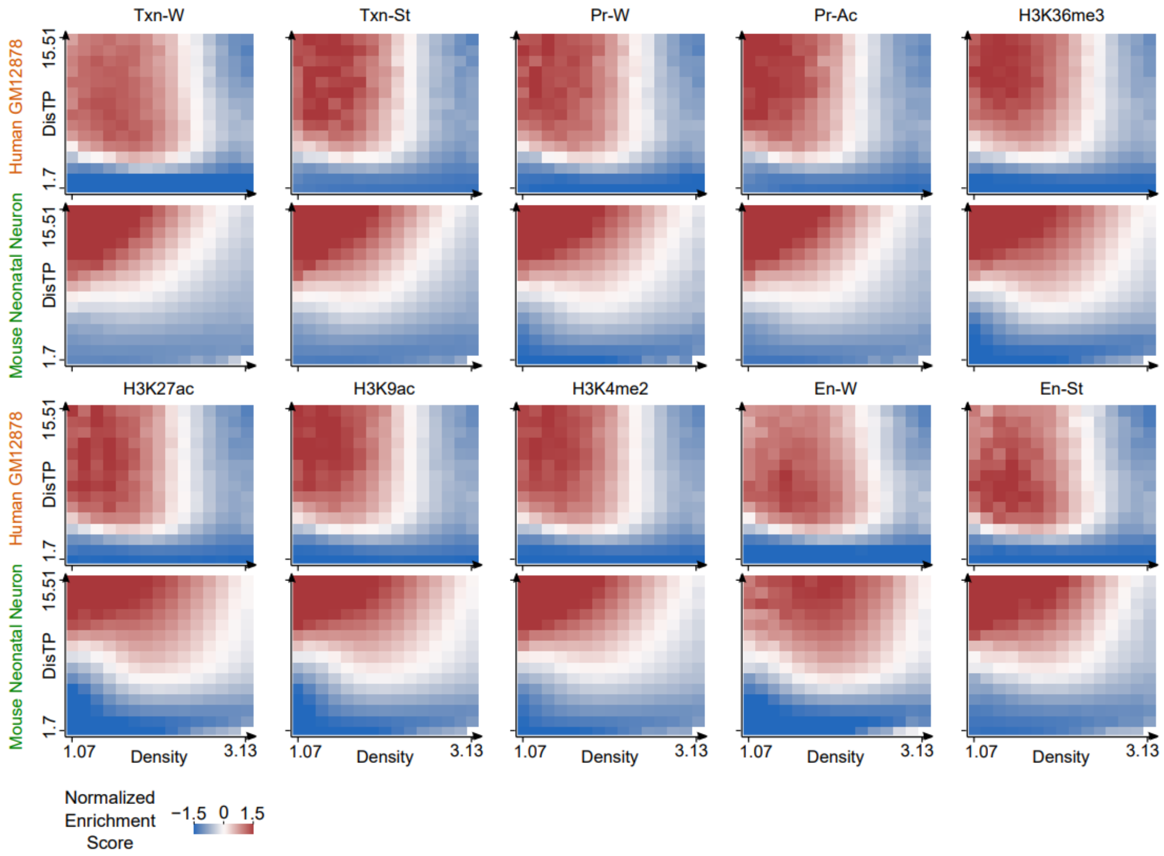

Figure S8. Enrichment scores of transcription-related markers on D^2 plot.
Weak transcription (Txn-W), strong transcription (Txn-St), weak promoter (Pr-W), active promoter (Pr-Ac), weak enhancer (En-W), strong enhancer (En-St) are chromatin states derived by HMM. Others are histone modifications detected by ChIP-seq.

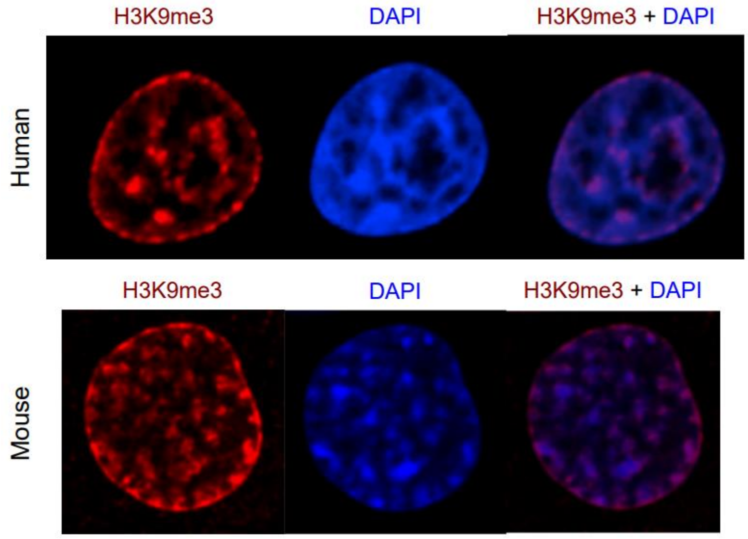

Figure S9. Human and mouse cell nuclei stained with DAPI (blue) and H3K9me3 (red)
Top two sub-panels are human Hela nuclei stained with anti-H3K9me3 (red) and DAPI (blue) from Poleshko et al.[13] Bottom sub-panels are mouse C2C12 nuclei stained with anti-H3K9me3 (red) and DAPI (blue) from Poleshko et al.[14] The red ring around the nuclear periphery showed that H3K9me3 enriched at nuclear periphery.

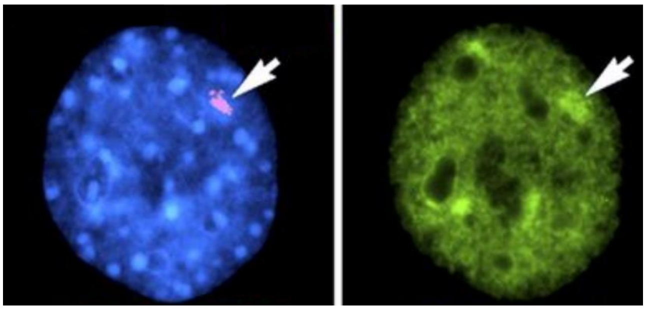

Figure S10. Mouse C2C12 cells stained with DAPI (blue) and anti-H3K27me3 (green), from Platt et al.[15]

High H3K27me3 signal regions and high DAPI signal regions co-localized at the nuclear interior, indicating that H3K27me3 enriched at inner high-density regions. All the cells of the above imaging experiments were in natural growth without special treatments.

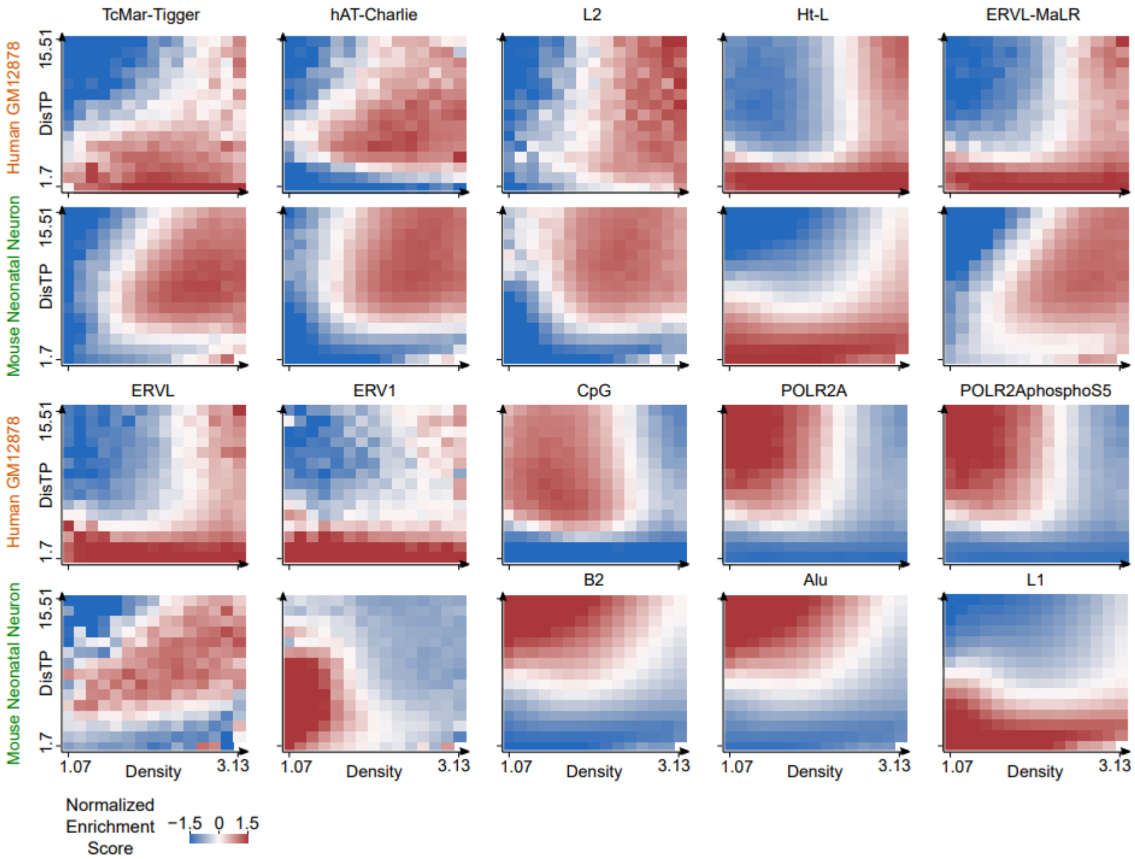

Figure S11. Enrichment scores of genetic and epigenetic markers on D^2 plot.
TcMar-Tigger belonged to Tc1 family of DNA transposon (class II transposon), length of which was between 1 and 5kb. hAT-Charlie belonged to hAT superfamily of DNA transposon (class II transposon), length of which was between 2.5 and 5kb. ERVL-MaLR, ERVL and ERV1 belonged to long terminal repeat (LTR), while L1, L2 belonged to long interspersed repetitive element (LINE). B2 and Alu belonged to short interspersed repetitive element (SINE). Low heterochromatin (Ht-L) was the chromatin state where no functional markers were enriched. CpG marked the percentage of CG sites. POLR2A and POLR2AphosphoS5 are detected by ChIP-seq.

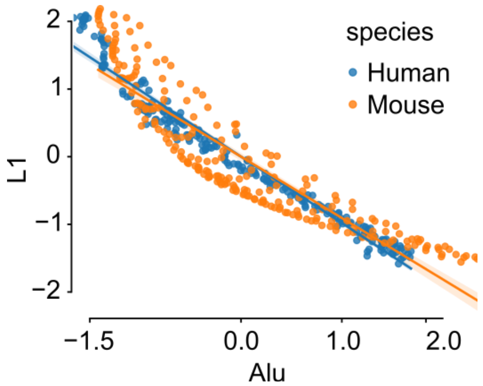

Figure S12. Scatter plot between fold changes of L1 and Alu.
The correlation was computed by Pearson Correlation (n=225 for human and n=224 for mouse). The colored lines denoted the fitting line of linear regression. Human PCC: -98.03%, *p*-value: 4.5×10^{-159}; Mouse PCC: -90.60%, *p*-value: 3.1×10^{-85}.

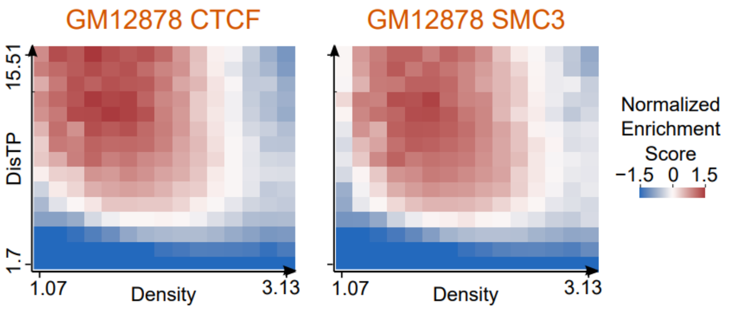

Figure S13. Enrichment scores of CTCF and SMC3 (sub-unit of cohesin) of human GM12878 cells on D^2 plot.

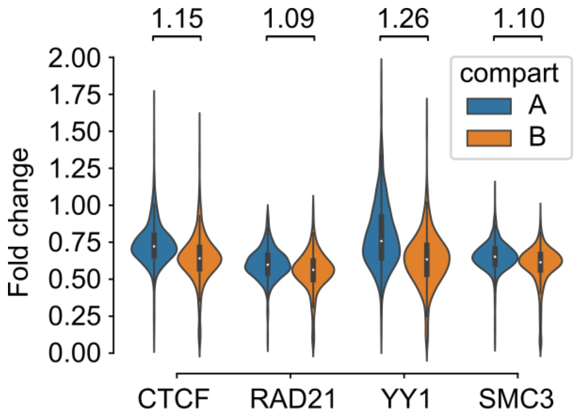

Figure S14. Box plots of enrichments of architectural proteins (CTCF, YY1, RAD21 and SMC3) at compartment A and compartment B.
The ratios of compartment A versus B were computed by the division of median value of enrichments at A to B.

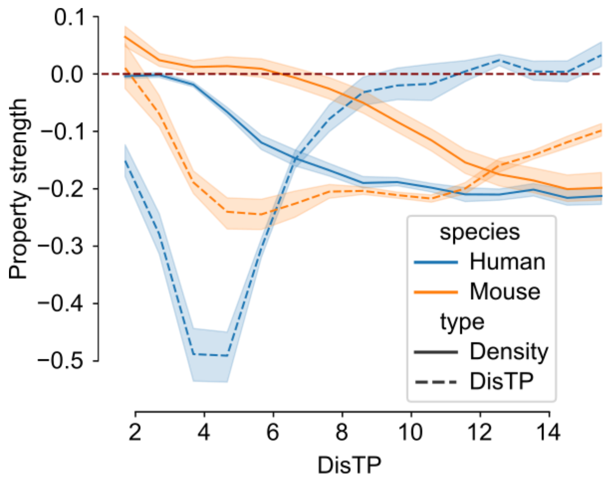

Figure S15. Property strength of density and DisTP at different DisTP.
Detailed method for computing the property strength was shown at Supplementary Methods. The positive values denoted positive correlation between property and transcription, while the negative values denoted negative correlation. The absolute value denoted the strength of correlation.

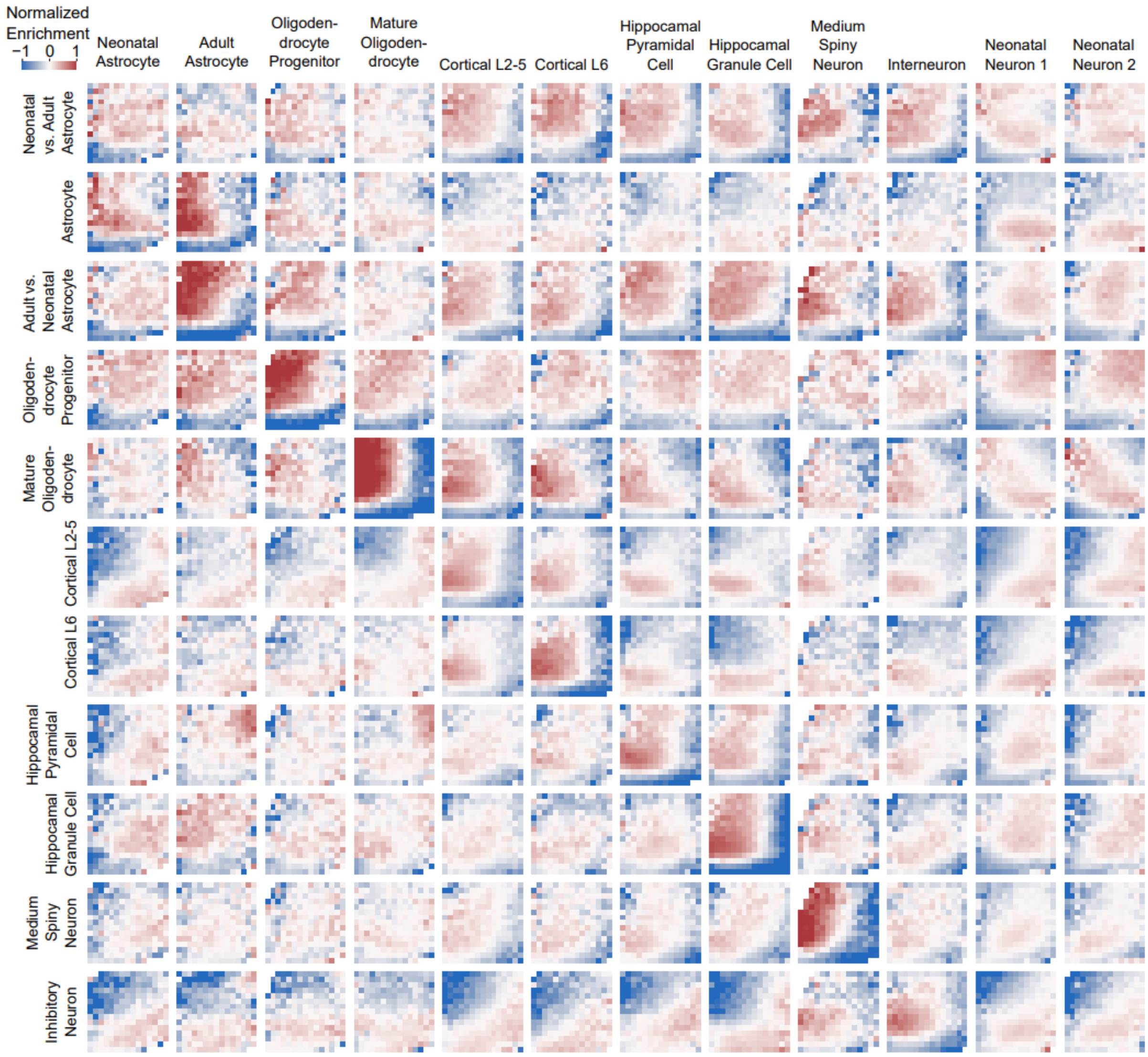

Figure S16. Enrichment scores of the lineage-specific active genes at different cell types of mouse brain cells.

The gene sets of enrichment scores from a given row were the same and labeled as row name. The cell types of enrichment scores from a given column were the same and labeled as column name. The cell-type-specific genes in the matching cell types were displayed in the D^2 plots with the same row and column name. The enrichment of blank physical states were not shown owing to a small number (n<200) of genomic bins.

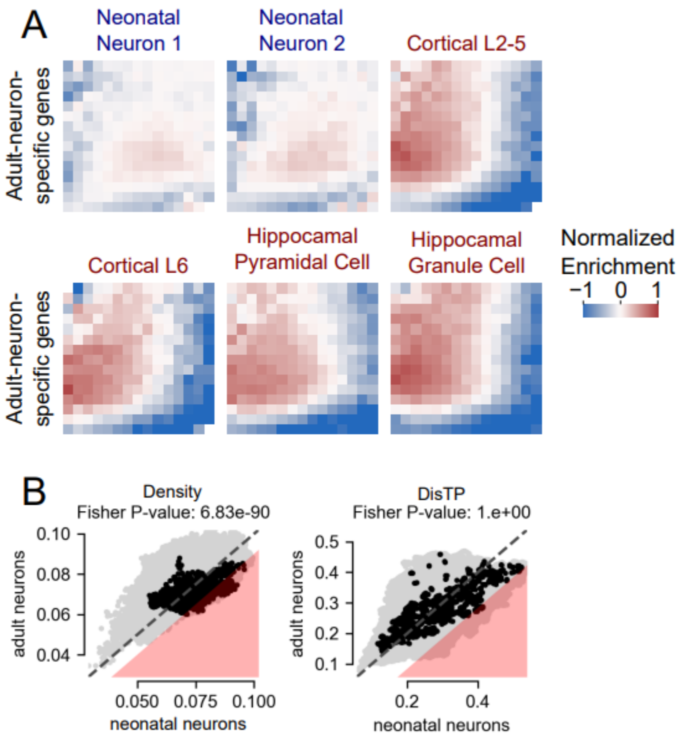

Figure S17. Movement of adult-neuron genes on D^2 plot.
(A) Enrichment scores of the adult-neuron genes at different cell types of mouse brain cells. The labels of neonatal neuron cell types were colored as blue, while adult neurons as red. **(B)** Comparison of density and DisTP between neonatal and adult neurons (n = 347 versus 363 cells). The *p*-values were computed by the two-sided Fisher's exact test.

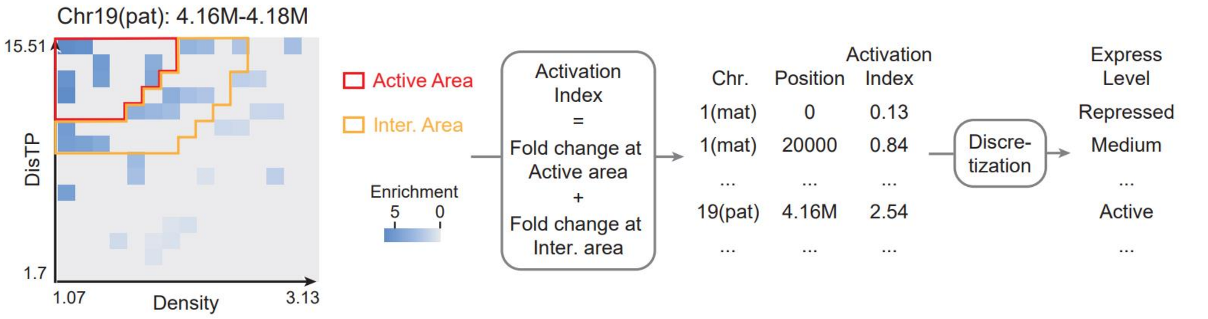

Figure S18. Illustration of computing activation index for defining the three transcriptional modes.

The left sub-panel showed an example genomic segment (parental haplotype of chr19: 4.16M-4.18M) on D^2 plot. The red lines marked the boundaries of active area, while the yellow ones marked the intermediate area. By discretization, we grouped the genome regions of a given cell type into repressed, medium and active expressing states. We then derived the three modes based on the changes of expressing states across cell types. Detailed methods were shown in Methods.

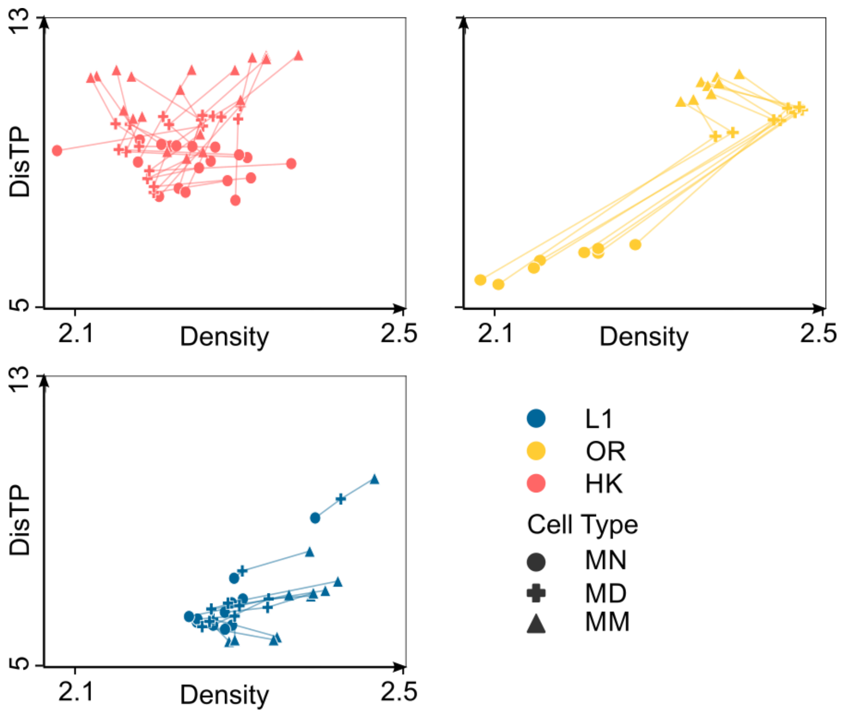

Figure S19. Movements of representative functional bins across three different developing stages of OSNs, for HK genes (left), ORs (Middle) or L1s (right), on D^2 plot. The functional elements should dominate and constitute at least 20% length of a given genomic bin to label it as elementary bin. The bins with two or more elementary labels were eliminated.

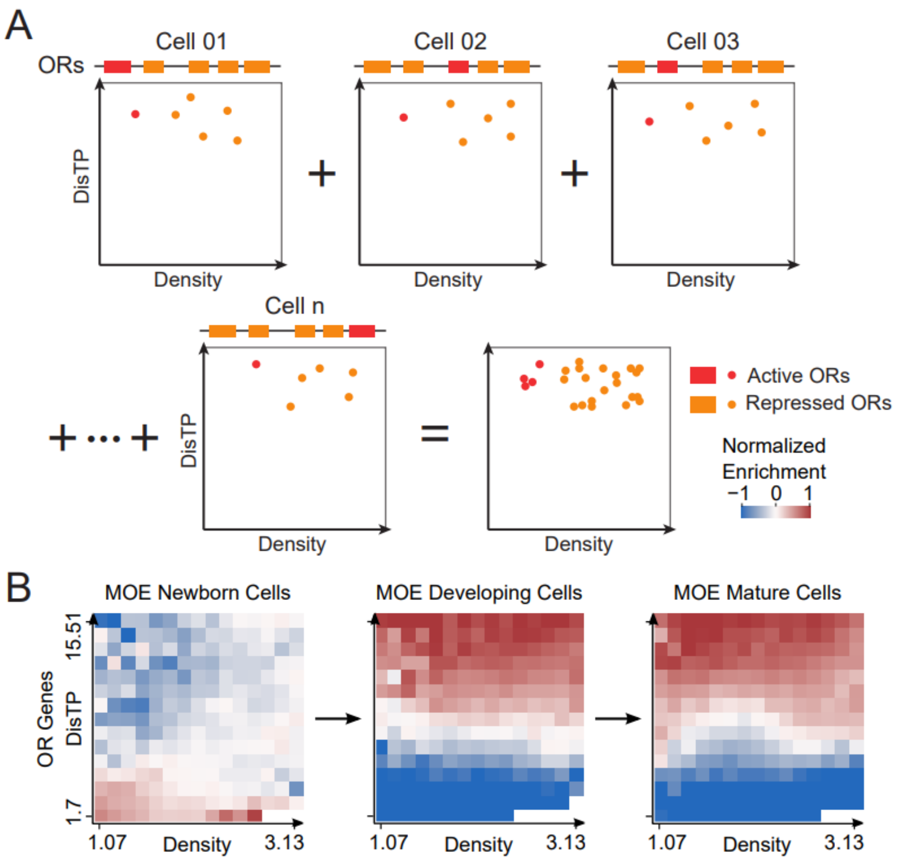

Figure S20. D^2 plots of ORs at three developing stages of OSNs.
(A) Visual explanation of featured distribution of ORs on D^2 plot of mature OSNs. For each cell, we draw a illustrated genome of five ORs (one of them is activated while others are repressed) at the top, and also their locations on D^2 plot at the bottom. By concatenating these cells, we draw the expected pattern at the bottom row.
(B) Real distribution of ORs on D^2 plot of three stages of OSNs. The enrichment scores of blank physical states were not shown owing to a small number (n<200) of genomic bins.

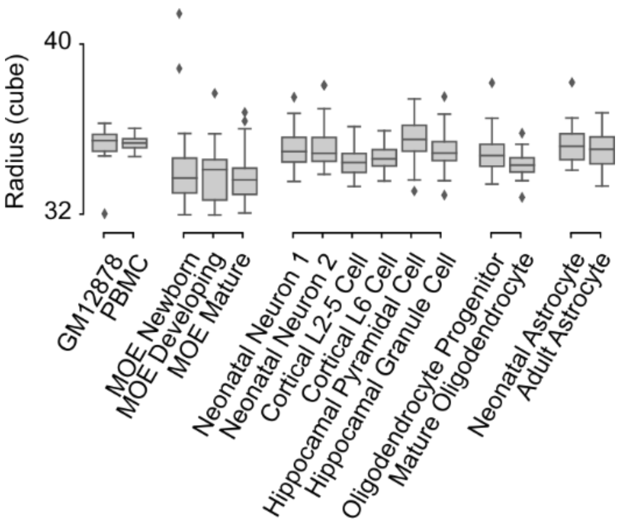

Figure S21. Nuclear radius of different cell types after normalization.
The cell types from the same cell differentiating lineages were clustered. Owing to that we put the genome structures in a 3D mesh, the original spatial coordinates are transformed into the mesh coordinates. Therefore, the unit of radius are number of cubes.

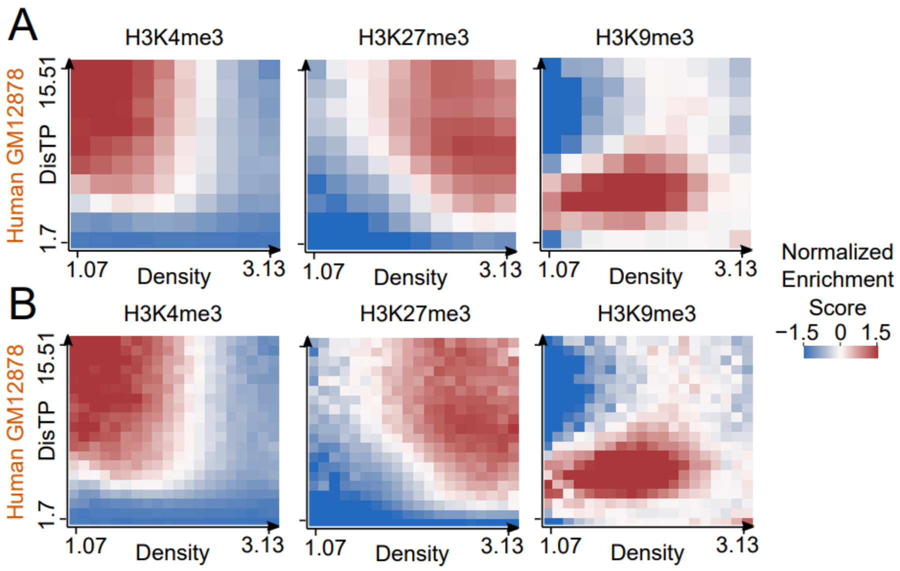

Figure S22. The impact of different numbers of physical states on enrichment analysis of D^2 plot.

The number of states was set as 225 (15*15) for the main text. Here the number of states on the left three patterns was set as 100 (10*10), while 400 (20*20) on the right. The observed patterns were similar to Figure 3B-C, indicating that the number of bins does not affect the enrichment patterns.

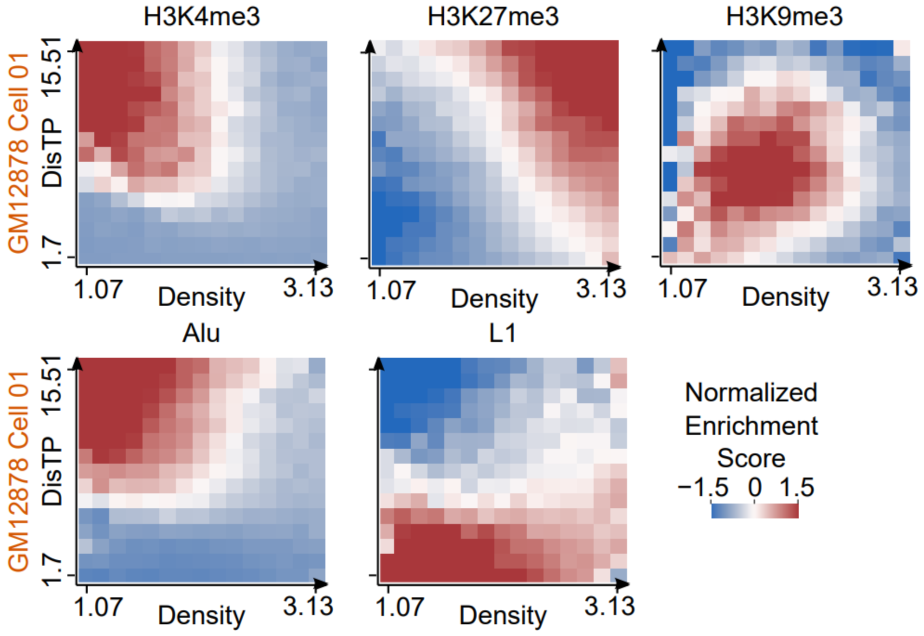

Figure S23. Enrichment scores drawn from mean values of density and DisTP.
The density and DisTP of a given genomic bin here were averaged first. The patterns were similar to Figure 3B-D, indicating that the averaging step did not affect the enrichment patterns.

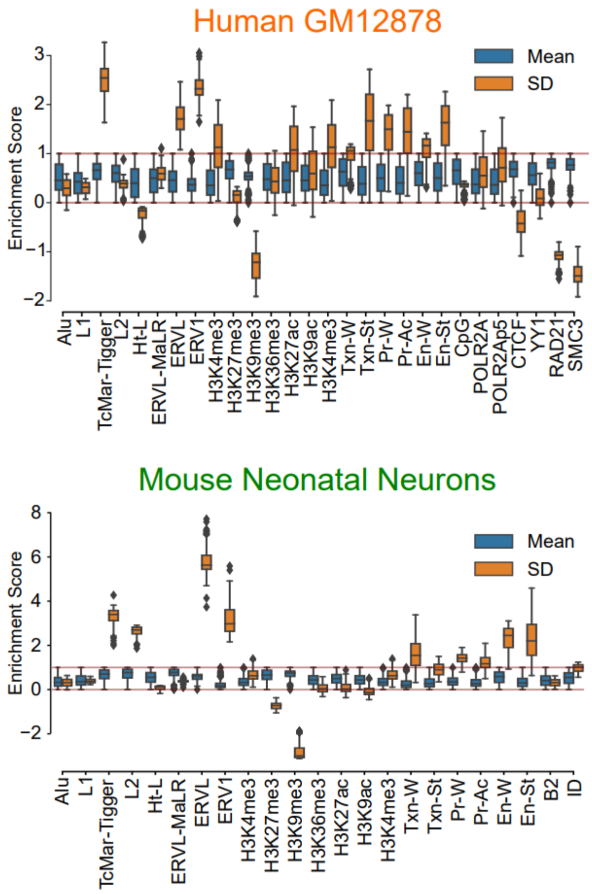

Figure S24. Both Mean and SD of genetic and epigenetic marker enrichment scores.
The max-min normalization on mean values was utilized for comparison between markers. This normalization was also used on SD values. Therefore, markers with SD less than 0 denoted low stochasticity, while others with higher than 1 denoted high stochasticity. The horizontal red lines denoted the maximum value (1) and minimum value (0) of mean value after max-min normalization. The comparison between mean and SD showed the extent of stochasticity.

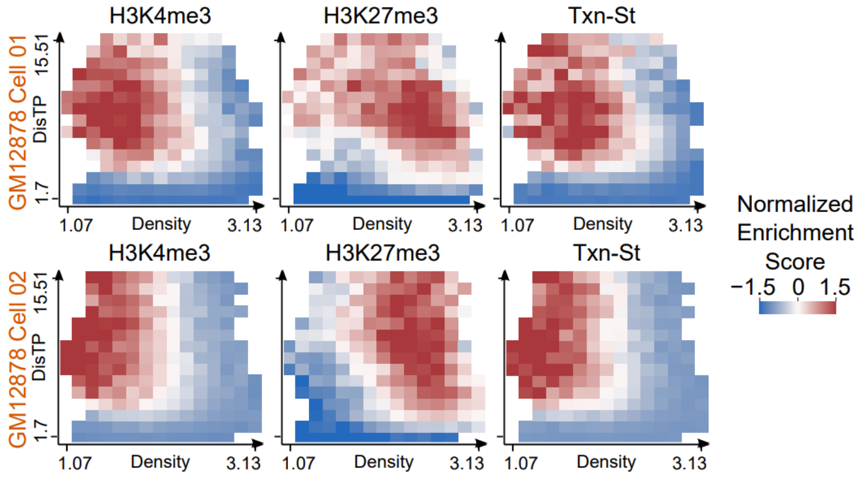

Figure S25. Enrichment scores of H3K4me3, H3K27me3 and Txn-St drawn from one cell.
The enrichment scores of blank physical states were not shown owing to a small number (n<200) of genomic bins. The enrichment scores of bulk cells were shown in Figure 3, S8 and S9.

References

[1] T. J. Stevens, D. Lando, S. Basu, L. P. Atkinson, Y. Cao, S. F. Lee, M. Leeb, K. J. Wohlfahrt, W. Boucher, A. O'Shaughnessy-Kirwan, J. Cramard, A. J. Faure, M. Ralser, E. Blanco, L. Morey, M. Sansó, M. G. S. Palayret, B. Lehner, L. di Croce, A. Wutz, B. Hendrich, D. Klenerman, E. D. Laue, *Nature* **2017**, *544*, 59.

[2] L. Tan, D. Xing, C. H. Chang, H. Li, X. S. Xie, *Science (1979)* **2018**, *361*, 924.

[3] A. Lesne, J. Riposo, P. Roger, A. Cournac, J. Mozziconacci, *Nature Methods* **2014**, *11*, 1141.

[4] J. Paulsen, O. Gramstad, P. Collas, *PLOS Computational Biology* **2015**, *11*, e1004396.

[5] H. Zhu, Z. Wang, *Bioinformatics* **2019**, *35*, 3981.

[6] L. Meng, C. Wang, Y. Shi, Q. Luo, *Nature Communications* **2021**, *12*, 4369.

[7] I. Solovei, A. S. Wang, K. Thanisch, C. S. Schmidt, S. Krebs, M. Zwerger, T. v. Cohen, D. Devys, R. Foisner, L. Peichl, H. Herrmann, H. Blum, D. Engelkamp, C. L. Stewart, H. Leonhardt, B. Joffe, *Cell* **2013**, *152*, 584.

[8] L. Tan, D. Xing, N. Daley, X. S. Xie, *Nature Structural and Molecular Biology* **2019**, *26*, 297.

[9] B. van Steensel, A. S. Belmont, *Cell* **2017**, *169*, 780.

[10] L. Tan, W. Ma, H. Wu, Y. Zheng, D. Xing, R. Chen, X. Li, N. Daley, K. Deisseroth, X. S. Xie, *Cell* **2021**, *184*, 741.

[11] Y. Wang, Z. Huang, K. Hu, J. Peng, W. Yao, W. Deng, J. Zuo, Y. Zhang, D. Yin, *Acta Biochimica et Biophysica Sinica* **2020**, DOI 10.1093/abbs/gmaa137.

[12] I. Solovei, K. Thanisch, Y. Feodorova, *Current Opinion in Cell Biology* **2016**, *40*, 47.

[13] A. Poleshko, K. M. Mansfield, C. C. Burlingame, M. D. Andrake, N. R. Shah, R. A. Katz, *Cell Reports* **2013**, *5*, 292.

[14] A. Poleshko, C. L. Smith, S. C. Nguyen, P. Sivaramakrishnan, K. G. Wong, J. I. Murray, M. Lakadamyali, E. F. Joyce, R. Jain, J. A. Epstein, *Elife* **2019**, *8*, DOI 10.7554/eLife.49278.

[15] E. J. Platt, L. Smith, M. J. Thayer, *Journal of Cell Biology* **2018**, *217*, 541.
